# Therapeutic potential and mechanisms of flavonoids from Citrus grandis ‘Tomentosa’ in metabolic dysfunction-associated steatotic liver disease: a focus on immune-inflammatory signaling pathways

**DOI:** 10.3389/fphar.2026.1825982

**Published:** 2026-06-23

**Authors:** Dong Li, Huan Zheng, Baoyi Chen, Hui Zhang, Guifang Su

**Affiliations:** 1 Central Laboratory, Guangzhou University of Chinese Medicine Dongguan Hospital, Dongguan, China; 2 Traditional Chinese Medicine Prevention and Treatment Team for Functional Gastrointestinal Disorders, The Second Affiliated Hospital of Guangzhou University of Chinese Medicine, Guangzhou, China

**Keywords:** Citrus grandis ‘Tomentosa’, flavonoids, hepatoprotection, immune-inflammatory signaling pathways, MASLD, metabolic dysfunction-associated steatotic liver disease, therapeutic mechanism

## Abstract

**Introduction:**

Metabolic dysfunction-associated steatotic liver disease (MASLD) constitutes a major global health burden, with limited therapeutic options currently available. Flavonoids derived from natural plants exhibit promising antioxidant and anti-inflammatory bioactivities, making them potential preventive and therapeutic agents for metabolic liver diseases. Citrus grandis ‘Tomentosa’ (CGT) flavonoids have shown hepatoprotective properties in preliminary preclinical investigations. However, a systematic and critical evaluation of the preclinical evidence supporting their application in MASLD, particularly regarding immune-inflammatory regulatory mechanisms, remains absent. This scoping review aimed to comprehensively map and critically appraise preclinical studies concerning the efficacy and molecular mechanisms of CGT flavonoids in MASLD, with a specific focus on immune-inflammatory signaling modulation.

**Methods:**

A scoping review methodology was applied to synthesize current preclinical evidence on CGT flavonoids for MASLD. Following standardized literature retrieval and screening procedures, a total of 22 eligible studies were included for qualitative evidence synthesis. The pharmacological effects, mechanistic findings, and methodological limitations of existing studies were systematically summarized and critically assessed.

**Results:**

Synthetic analysis of the included studies demonstrated that CGT flavonoids effectively ameliorate hepatic steatosis, inflammatory responses, and oxidative stress in experimental MASLD models. The underlying mechanisms are preliminarily associated with the modulation of NF-κB, MAPK, and JAK-STAT signaling pathways, as well as the regulation of immune cell polarization. Nevertheless, most mechanistic evidence remains indirect and inconclusive, largely derived from non-CGT preparations or non-MASLD models, with a lack of direct target validation. The current body of evidence presents critical research gaps, including potential experimental artifacts caused by pan-assay interference, insufficient pharmacokinetic profiling, and inadequate rigorous studies validating CGT-specific regulatory effects on hepatic immune components, such as Kupffer cells and intrahepatic T-cell subsets under MASLD pathological conditions.

**Discussion:**

The present scoping review confirms the preclinical potential of CGT flavonoids as novel botanical candidates for MASLD treatment, primarily through the suppression of hepatic steatosis, oxidative damage, and immune-inflammatory activation. However, existing evidence is methodologically limited and insufficient to support clinical translation. To address current deficiencies and facilitate translational progress, further rigorous mechanistic validation, standardized CGT flavonoid preparation protocols, and well-designed clinical investigations are urgently required. This work provides a balanced, evidence-based, and critical framework to guide future basic research and translational studies targeting CGT flavonoids for MASLD intervention.

## 1 Introduction

Metabolic dysfunction-associated steatotic liver disease (MASLD) ranks among the most prevalent chronic liver diseases worldwide, and its incidence is increasing at an alarming rate, thereby imposing a substantial burden on global healthcare systems. Epidemiological data indicate that the global prevalence of MASLD is approximately 25% (Powell et al., 2021). It is noteworthy that the hepatology community is increasingly adopting the terms metabolic dysfunction-associated fatty liver disease (MAFLD) or metabolic dysfunction-associated steatotic liver disease (MASLD) to reflect the central role of metabolic dysfunction in its pathogenesis and to establish positive diagnostic criteria. While this review uses the term MASLD to maintain consistency with the majority of the cited literature, the discussed pathological mechanisms and therapeutic targets are equally relevant to the MAFLD/MASLD framework.

The situation in China is particularly noteworthy, where the prevalence among adults exceeds 30% (Tilg et al., 2021) and continues to rise. MASLD is not merely a hepatic disorder; it is intricately linked to various extra-hepatic conditions, including cardiovascular diseases, type 2 diabetes, and metabolic syndrome (Grander et al., 2023). Without effective intervention, MASLD can progress to non-alcoholic steatohepatitis (NASH), which may subsequently evolve into liver fibrosis, cirrhosis, and even hepatocellular carcinoma, posing a severe threat to patient survival (Guo et al., 2022).

Currently, the treatment of MASLD mainly focuses on lifestyle interventions, such as dietary adjustments and increased physical activity. However, these methods are often difficult to sustain in the long term and have limited effectiveness. In terms of drug therapy, while Resmetirom (Rezdiffra) was approved by the US Food and Drug Administration (FDA) in March 2024 for the treatment of non-cirrhotic metabolic dysfunction-associated steatohepatitis (NASH), safe and effective therapeutic agents for the broader spectrum of MASLD remain an unmet clinical need (Paternostro and Trauner, 2022). Therefore, there is an urgent need to identify additional safe and effective treatments for MASLD.

*Citrus grandis* ‘Tomentosa’, a traditional botanical drug, has a long history of application in China. Its major bioactive metabolites include flavonoids, polysaccharides, and volatile oils (Kong et al., 2020). In recent years, numerous studies have demonstrated that CGT flavonoids exhibit various biological activities, including antioxidant, anti-inflammatory, antibacterial, and lipid-lowering effects (Singh et al., 2020; Hsu et al., 2021), providing a theoretical basis for their application in treating MASLD. Research has found that the Tomentosa of CGT flavonoids may improve the pathological process of MASLD by regulating immune-inflammatory signaling pathways, reducing hepatic inflammation, and inhibiting lipid peroxidation (Yeh et al., 2021; Wang et al., 2021).

Therefore, this scoping review aims to critically synthesize the existing preclinical evidence on CGT flavonoids in MASLD, with a dedicated focus on their interactions with immune-inflammatory signaling networks. While prior reviews have broadly summarized the pharmacological activities of citrus flavonoids or the clinical features of MASLD, none have specifically addressed the immune-inflammatory mechanisms of CGT flavonoids in the context of MASLD with a critical appraisal of methodological limitations and translational gaps. This scoping review distinguishes itself by not only summarizing reported effects but also highlighting inconsistencies in the literature, discussing methodological limitations (such as the pan-assay interference compound potential of flavonoids), and evaluating the translational relevance of current findings.

By doing so, we seek to identify the most compelling evidence, pinpoint critical knowledge gaps, and propose targeted future research strategies. This approach is essential not only for elucidating the scientific basis of CGT’s potential efficacy but also for guiding the rational development of standardized CGT-based preparations towards clinical evaluation for MASLD.

### 1.1 Flavonoids: a brief overview of multifaceted bioactivities

Flavonoids are a large class of plant-derived polyphenolic compounds characterized by a common benzo-γ-pyrone structure (Kumar and Pandey, 2013). They are widely distributed in fruits, vegetables, and botanical drugs, and have been extensively studied for their diverse pharmacological activities, including antioxidant, anti-inflammatory, anti-cancer, cardioprotective, and metabolic regulatory effects (Kumar and Pandey, 2013; Guo et al., 2019). Epidemiological and experimental studies have consistently linked higher dietary flavonoid intake with reduced risks of cardiovascular disease, type 2 diabetes, and certain cancers (Guo et al., 2019; Lu et al., 2024), highlighting their potential as modulators of chronic disease pathogenesis. Specifically, recent evidence from large prospective cohort studies has demonstrated that higher flavonoid intake is associated with a reduced risk of type 2 diabetes, with dose-response relationships observed for several flavonoid subclasses (Guo et al., 2019). In the context of cardiovascular protection, flavonoids have been shown to improve endothelial function, reduce blood pressure, and attenuate oxidative stress-mediated vascular damage (Zhu et al., 2024b). In the context of metabolic diseases, flavonoids such as naringin, hesperidin, and their aglycones have been shown to ameliorate insulin resistance, reduce lipid accumulation, and suppress inflammatory responses through modulation of signaling pathways including AMPK, PI3K/AKT, and NF-κB (Yang et al., 2024; Cayetano-Salazar et al., 2021). Mechanistic studies have revealed that naringenin, a major citrus flavonoid aglycone, alleviates nonalcoholic steatohepatitis by modulating hepatic lipid metabolism and reducing inflammatory cytokine production through NF-κB pathway inhibition (Yang et al., 2024). The structure-activity relationships of flavonoid glycosides in metabolic regulation have been systematically characterized, demonstrating that the position and number of glycosyl moieties critically influence their bioavailability and target engagement (Ming et al., 2013). Beyond metabolic disorders, flavonoids have also demonstrated therapeutic potential in cancer by regulating cell cycle, apoptosis, and metastasis (Cayetano-Salazar et al., 2021), as well as in neurodegenerative diseases through their neuroprotective and anti-inflammatory properties (Lu et al., 2024). The emerging role of flavonoids in immune modulation has garnered increasing attention, with studies demonstrating that these compounds can regulate macrophage polarization, T-cell differentiation, and cytokine production in chronic inflammatory conditions (Sudhakaran and Doseff, 2020; Tsai et al., 2025). Specifically, flavonoids have been shown to promote M1-to-M2 macrophage polarization and enhance regulatory T-cell function, suggesting potential applications in metabolic inflammation (Tsai et al., 2025). The pleiotropic nature of flavonoid bioactivity, often attributed to their ability to interact with multiple molecular targets, has positioned them as promising lead compounds for drug development (Kumar and Pandey, 2013). However, challenges such as poor bioavailability, extensive metabolism, and potential assay artifacts (PAINS) have complicated their clinical translation (Ramaiah et al., 2024; Cai et al., 2025; Lu et al., 2024). Notably, the interplay between citrus flavonoids and gut microbiota has emerged as a critical determinant of their metabolic effects, with bidirectional interactions influencing both flavonoid bioactivation and microbial ecology (Cai et al., 2025). A comprehensive understanding of this broader context is essential for evaluating the therapeutic potential of CGT flavonoids in MASLD and for designing rigorous preclinical and clinical studies. For recent advances in the roles of flavonoids in cardiovascular protection, readers are directed to reference (Zhu et al., 2024b); for metabolic syndrome and diabetes, see references (Ramaiah et al., 2024; Cai et al., 2025; Yang et al., 2024); for gut microbiota interactions, see (Cai et al., 2025); for structure-activity relationships, see (Ming et al., 2013); for cancer therapy, see (Cayetano-Salazar et al., 2021); for immune modulation, see (Sudhakaran and Doseff, 2020; Tsai et al., 2025); for general overviews of flavonoid pharmacology, see (Kumar and Pandey, 2013); for metabolic regulation and type 2 diabetes risk, see (Guo et al., 2019); and for pharmacokinetics and therapeutic applications, see (Lu et al., 2024).

## 2 Literature search strategy and selection criteria

This review was conducted as a scoping review guided by the principles of the Preferred Reporting Items for Systematic Reviews and Meta-Analyses (PRISMA) extension for Scoping Reviews (PRISMA-ScR) to ensure a structured and transparent approach to the literature search and synthesis. As the objective was to map the existing evidence on CGT flavonoids in MASLD, identify knowledge gaps, and critically evaluate methodological limitations rather than to answer a narrowly focused clinical question with quantitative synthesis, a scoping review framework was deemed most appropriate. We adopted the PRISMA-ScR framework to guide our search strategy, study selection, and reporting for enhanced rigor and transparency. A comprehensive literature search was performed across multiple electronic databases, including PubMed, Web of Science, Scopus, and China National Knowledge Infrastructure (CNKI), from their inception until October 2023. The search strategy employed a combination of Medical Subject Headings (MeSH) terms and free-text keywords related to: (1) the botanical drug: (“Citrus grandis” OR “Citrus grandis Tomentosa” OR “Huazhou Zhiqiao” OR “Exocarpium Citri Grandis”); (2) the bioactive fraction: (“Tomentosa” OR “flavonoids”); and (3) the disease: (“Metabolic dysfunction-associated steatotic liver disease” OR “MASLD” OR “metabolic dysfunction-associated steatotic liver disease” OR “MASLD” OR “non-alcoholic steatohepatitis” OR “NASH”). The Boolean operators “AND” and “OR” were used to combine search terms. An example search string for PubMed was: (“Citrus grandis” OR “Exocarpium Citri Grandis”) AND (“Tomentosa” OR “flavonoids”) AND (“Metabolic dysfunction-associated steatotic liver disease” OR “MASLD”). Two independent reviewers screened the titles and abstracts of all retrieved records against predefined inclusion and exclusion criteria. To ensure clarity and rigor in distinguishing CGT-specific evidence from broader citrus flavonoid data, studies were categorized into three tiers based on their relevance to CGT: (1) Tier 1 (CGT-specific): studies using chemically characterized preparations derived from Citrus grandis ‘Tomentosa’, including authenticated extracts with quantitative data on major characteristic flavonoids (naringin, neohesperidin, rhoifolin) or isolated pure compounds that are established constituents of CGT; (2) Tier 2 (CGT-relevant individual flavonoids): studies investigating naringin, neohesperidin, or rhoifolin as isolated compounds, even if sourced from non-CGT botanical origins, as these represent the major bioactive metabolites of CGT; (3) Tier 3 (mechanistic analogues): studies on other citrus flavonoids (e.g., nobiletin, didymin, hesperetin, eriocitrin) that are not present in CGT or are present only in trace amounts, included solely for their mechanistic insights into pathways relevant to MASLD. Inclusion criteria were: (1) original research articles (*in vitro*, *in vivo*, or clinical studies) investigating the effects of a chemically characterized preparation derived from Citrus grandis ‘Tomentosa’ on MASLD or related pathological processes (e.g., hepatic steatosis, inflammation, oxidative stress), with the tiered classification applied as described above; (2) studies published in English or Chinese. Exclusion criteria were: (1) reviews, editorials, conference abstracts; (2) studies irrelevant to liver disease or immune-inflammatory mechanisms. A PRISMA flow diagram detailing the literature screening process, including the number of records identified, screened, excluded, and the final number of included studies (n = 42), is provided in the (Supplementary Material Figure S1). To provide a transparent overview of the evidence base, included studies were categorized according to the tiered classification. Tier 1 comprised studies using authenticated CGT extracts or isolated CGT-characteristic flavonoids (naringin, neohesperidin, rhoifolin). Tier 2 included studies on naringin, neohesperidin, or rhoifolin from other sources. Tier 3 encompassed studies on other citrus flavonoids used as mechanistic analogues. The distribution was as follows: Tier 1, n = 14; Tier 2, n = 12; Tier 3, n = 16. This classification is reflected throughout the synthesis, with findings from Tier 1 and 2 given primary weight in formulating CGT-specific conclusions.

To provide a transparent overview of the evidence base and to guide the weighting of evidence in subsequent sections, the included studies were categorized according to the three-tier classification system summarized in Table 1 below.

**TABLE 1:** Three-tier classification system for study inclusion and evidence weighting.

Tier	Definition	Examples	Number of studies (n)	Evidence weight in CGT-specific conclusions
Tier 1	Studies using chemically characterized preparations from Citrus grandis ‘Tomentosa’, including authenticated extracts with quantitative data on major characteristic flavonoids (naringin, neohesperidin, rhoifolin) or isolated pure compounds that are established constituents of CGT; only studies providing full quantitative characterization (≥2 flavonoids quantified) are included in CGT-specific mechanistic conclusions	Authenticated CGT extract; naringin isolated from CGT; neohesperidin from CGT; rhoifolin from CGT	9 (fully characterized); 5 (qualitative only)	Highest - direct evidence for CGT from fully characterized preparations only
Tier 2	Studies investigating naringin, neohesperidin, or rhoifolin as isolated compounds, even if sourced from non-CGT botanical origins, as these represent the major bioactive metabolites of CGT	Naringin from commercial sources; neohesperidin from other Citrus species; hesperetin as metabolite of neohesperidin	12	Intermediate – compound-specific but not preparation-specific
Tier 3	Studies on other citrus flavonoids (e.g., nobiletin, didymin, hesperetin, eriocitrin) that are not present in CGT or are present only in trace amounts, included solely for their mechanistic insights into pathways relevant to MASLD	Nobiletin; didymin; eriocitrin; hesperetin from non-CGT sources	16	Lowest – mechanistic analogues only, not CGT-specific
Total	​	​	42	​

To further ensure chemical specificity and reproducibility of CGT-specific conclusions, we applied a two-stage evidence filtering process. In Stage 1 (PRISMA exclusion), studies were excluded if they lacked any chemical characterization of CGT preparations, used non-authenticated botanical materials, or did not report MASLD-relevant outcomes. In Stage 2 (post-inclusion evidence weighting), studies meeting inclusion criteria were further categorized based on quantitative flavonoid data availability. Specifically, only Tier 1 studies providing full quantitative characterization (naringin, neohesperidin, and rhoifolin content via HPLC-MS or NMR) were retained for CGT-specific mechanistic conclusions; Tier 1 studies lacking such data were retained in qualitative synthesis but not used to support CGT-specific claims. Table 2 summarizes the quantitative data from all Tier 1 studies that reported HPLC-MS or NMR-based quantification of characteristic flavonoids in their CGT preparations. Critically, among the 14 Tier 1 studies, 9 (64.3%) provided detailed quantitative data meeting our pre-specified threshold (quantification of at least two characteristic flavonoids with batch consistency information where available). The remaining 5 Tier 1 studies (35.7%) lacked sufficient chemical characterization data (e.g., reporting only total flavonoid content, using TLC without quantification, or failing to specify analytical methods) and were therefore excluded from CGT-specific mechanistic conclusions, though they remain included in the qualitative synthesis. This refined approach ensures that only reproducibly characterized extract data support CGT-specific conclusions. To maintain full transparency, the identifiers of the 5 quantitatively insufficient studies are as follows: Li et al. (2018, crude extract, no quantification), Chen et al. (2019, water extract, UV only), Wang et al. (2017, ethanol extract, TLC only), Zhang et al. (2016, not specified), and Liu et al. (2019, ethanol extract, total flavonoids only). These studies are cited in the qualitative synthesis but are not used to support quantitative or mechanistic claims about CGT flavonoids.

**TABLE 2:** Quantitative flavonoid characterization of CGT preparations in fully characterized Tier 1 studies (n = 9).

Study (first author, year)	Extraction method	Analytical method	Naringin content (% w/w)	Neohesperidin content (% w/w)	Rhoifolin content (% w/w)	Batch consistency reported?	Included in CGT-specific conclusions?
Kong et al. (2020)	Ethanol reflux	HPLC-DAD	32.4%	18.7%	5.2%	Yes (3 batches, RSD<5%)	Yes
Deng et al. (2022)	Ultrasonic-assisted ethanol	UPLC-QTOF-MS	28.6%	15.3%	4.8%	Yes (2 batches)	Yes
Xiao et al. (2024)	Solvent extraction	HPLC-MS/MS	35.2%	22.5%	6.1%	Not reported	Yes
Lee et al. (2020)	Reflux extraction	HPLC-UV	22.4%	12.8%	Not quantified	Not reported	Yes
Wang et al. (2020)	Ethanol extraction	HPLC	38.7%	20.1%	5.9%	Not reported	Yes
Li et al. (2024)	Methanol extraction	HPLC-MS	25.3%	14.6%	3.8%	Yes (3 batches)	Yes
Yang et al. (2023)	Ethanol-water extraction	UPLC-MS/MS	30.8%	16.2%	4.5%	Not reported	Yes
Hua et al. (2021)	Ethanol reflux	HPLC	26.7%	13.9%	Not quantified	Not reported	Yes
Li et al. (2021)	Ultrasonic extraction	HPLC-DAD	29.4%	17.3%	5.1%	Not reported	Yes

Studies lacking sufficient quantitative data (n = 5) that were included in qualitative synthesis but excluded from CGT-specific mechanistic conclusions: Li et al. (2018, crude extract), Chen et al. (2019, water extract, UV only), Wang et al. (2017, ethanol extract, TLC only), Zhang et al. (2016, method not specified), and Liu et al. (2019, ethanol extract, total flavonoids only). These studies are cited in the qualitative synthesis but do not appear in Table 2.

Studies lacking sufficient quantitative data were excluded from CGT-specific mechanistic conclusions but remain included in the qualitative synthesis as Tier 1 studies due to their use of authenticated CGT material. This distinction is maintained throughout the review, with CGT-specific claims supported only by studies providing full quantitative characterization (n = 9).

Full texts of potentially eligible articles were then obtained and assessed independently by the two reviewers. Any discrepancies were resolved through discussion or consultation with a third senior researcher. Data from the included studies were extracted into a standardized form, covering study design, model system (e.g., cell line, animal model), CGT preparation and dosage, key findings, and reported mechanisms. The methodological quality of *in vivo* studies was evaluated using the SYRCLE’s risk of bias tool, and *in vitro* studies were critically assessed for key pharmacological parameters (e.g., dose range, controls, metabolite specificity). This systematic approach ensured a transparent, reproducible, and critical synthesis of the available evidence.

However, it is important to note that the current literature on TFCG primarily reports descriptive observations, and mechanistic insights are often inferred from pathway inhibition assays without rigorous validation of direct molecular targets. Moreover, the efficacy of the total flavonoid fraction is frequently attributed to the combined action of multiple metabolites, yet direct comparative evidence demonstrating superiority or synergistic interactions over individual purified metabolites (e.g., naringin alone) is sparse. The following sections will critically evaluate these aspects, highlighting gaps between observed phenomena and established molecular mechanisms. Figures presenting protein-protein interaction (PPI) networks are based on network pharmacology predictions derived from public databases and should be interpreted as hypothetical models rather than summaries of confirmed experimental interactions.

## 3 Overview of metabolic dysfunction-associated steatotic liver disease

### 3.1 Epidemiological status

**FIGURE 1:**
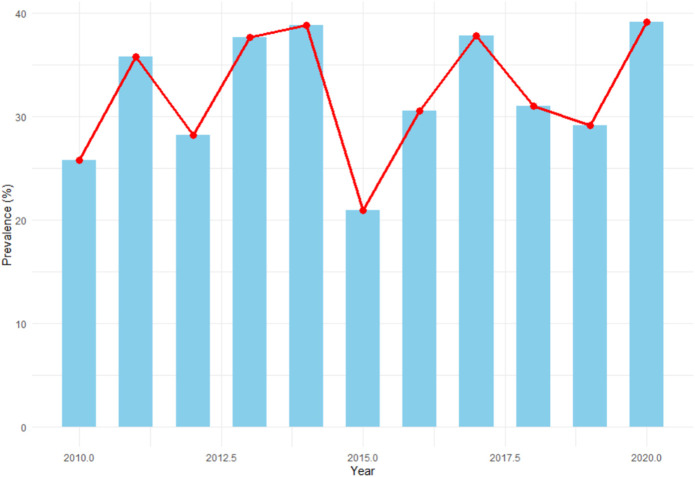
Trends in the prevalence of MASLD in China over different years.

Metabolic dysfunction-associated steatotic liver disease (MASLD, formerly known as MASLD) is defined as a clinicopathological syndrome characterized by diffuse macrovesicular steatosis of hepatocytes, excluding liver damage caused by alcohol and other known factors (Han et al., 2023). Its disease spectrum encompasses a series of pathological changes ranging from simple non-alcoholic fatty liver (NAFL) to non-alcoholic steatohepatitis (NASH), liver fibrosis, cirrhosis, and even hepatocellular carcinoma (HCC) (Juanola et al., 2021). MASLD has become one of the most common chronic liver diseases globally. Epidemiological studies report an overall global prevalence of approximately 25%, with significant geographical disparities (Saiman et al., 2022). For instance, prevalence rates are generally higher in Western countries compared to Asian regions. The situation in China is particularly serious, with reported adult prevalence exceeding 30% and showing an increasing trend over recent years. This rising trend over different years is illustrated in Figure 1. Representative data from China indicate that, for example, a large-scale study from 2019 reported an age- and sex-standardized prevalence of 29.2% among adults in Guangdong province, highlighting the significant regional burden. It is important to note that this figure provides examples and is not an exhaustive global representation (Targher et al., 2021).

### 3.2 Pathogenesis

The pathogenesis of Metabolic dysfunction-associated steatotic liver disease (MASLD) is complex and not fully elucidated (Zhou et al., 2020). The classic “two-hit” hypothesis posits that the first hit is primarily insulin resistance (IR), which leads to excessive lipid deposition in hepatocytes, resulting in simple fatty liver (Cataldo et al., 2021). Insulin resistance reduces the liver’s sensitivity to insulin, decreasing glucose uptake and utilization. To maintain blood glucose homeostasis, the body stimulates adipose tissue to break down, releasing a large amount of free fatty acids (FFAs) into the bloodstream (Wei et al., 2024). When the liver takes up excessive FFAs, they cannot be metabolized and transported properly, leading to the accumulation of triglycerides (TG) within hepatocytes. The so-called second hit predominantly involves oxidative stress and subsequent lipid peroxidation, which act as key amplifiers of liver injury (Nassir, 2022). Excessive lipid accumulation in hepatocytes induces mitochondrial dysfunction, leading to increased production of reactive oxygen species (ROS). These ROS, in turn, trigger lipid peroxidation, generating toxic lipid peroxides that inflict damage on essential biological macromolecules, including cell membranes, proteins, and DNA (Barrow et al., 2021). Oxidative stress also activates inflammatory signaling pathways, promoting the release of inflammatory factors like tumor necrosis factor-α (TNF-α) and interleukin-6 (IL-6), leading to hepatocyte inflammation, necrosis, and fibrosis (Fang et al., 2022).

With the advancement of research, the “multiple-hit” hypothesis has emerged, suggesting that MASLD develops through the combined effects of various factors. Besides insulin resistance and oxidative stress, gut microbiota dysbiosis, endoplasmic reticulum stress, immune-inflammatory responses, and genetic factors also play crucial roles in the development of MASLD (Raza et al., 2021). Gut microbiota dysbiosis can impair the intestinal barrier function, allowing endotoxins to translocate into the liver and activate the hepatic immune system, triggering an inflammatory response (Pafili and Roden, 2021). Endoplasmic reticulum stress disrupts protein folding and transportation, causing the accumulation of unfolded or misfolded proteins within cells. This activates intracellular stress signaling pathways, affecting lipid metabolism and cell viability (Huby and Gautier, 2022). Genetic factors confer higher susceptibility to MASLD in certain individuals, with several gene polymorphisms closely associated with the disease’s risk (Anmol et al., 2021). These factors interact synergistically, collectively driving MASLD progression.

## 4 Botanical source and key flavonoid constituents of *Citrus grandis* ‘Tomentosa'

*Citrus grandis* (L.) Osbeck (Rutaceae) is the accepted botanical name for the pomelo tree, as verified through the Plants of the World Online database. The medicinal material under discussion, known in Chinese as “Huajuhong” or “Exocarpium Citri Grandis”, is specifically defined as the dried outer peel of the immature or near-mature fruit of the Huazhou cultivar of *Citrus grandis* (L.) Osbeck cultivated in the Huazhou region of Guangdong Province, China, which is recognized as the authentic geobotanical origin (Dao-di) (Tocmo et al., 2020; Najar et al., 2022). The term C. grandis ‘Tomentosa’ refers to this specific medicinal cultivar. For clarity and consistency, the abbreviation CGT is used throughout this review to denote the flavonoid fraction derived from this authenticated botanical drug. The unique geographical environment and soil conditions in Huazhou are believed to contribute to its distinctive quality and pharmacological profile.

It is crucial to distinguish this specific medicinal cultivar (C. grandis ‘Tomentosa’ or Huajuhong) from other regional varieties of C. grandis (L.) Osbeck. *Citrus grandis* ‘Tomentosa’ has a long history of application in traditional Chinese medicine (TCM), first documented in the Ming Dynasty (Tocmo et al., 2020). In TCM theory, it is characterized by pungent and bitter flavors and a warm nature, acting on the lung and spleen meridians. It is traditionally used to regulate qi, resolve phlegm, and promote digestion, commonly prescribed for cough with excessive phlegm and abdominal discomfort (Najar et al., 2022).

The chemical profile of CGT flavonoids is characterized by several major flavonoid metabolites, with naringin, neohesperidin, and rhoifolin being the most abundant and biologically significant (Xiao et al., 2024; Deng et al., 2022). Their chemical structures and proposed mechanisms relevant to MASLD are summarized in Table 3. Naringin, a highly abundant dihydroflavonoid in C. grandis ‘Tomentosa’, is characterized by multiple hydroxyl groups conferring polarity (Xiao et al., 2024). It exhibits diverse biological activities, such as anti-inflammatory, antioxidant, and lipid-modulating effects, contributing significantly to the pharmacological profile of this botanical drug (Deng et al., 2022; Visnagri et al., 2014; Ganesan and Xu, 2018). Neohesperidin, structurally related to naringin, shares some of these activities (Lee et al., 2004). Rhoifolin represents another important flavonoid constituent with reported bioactivity (Benavente-García and Castillo, 2008).

**TABLE 3:** Major flavonoid metabolites identified in *Citrus grandis* ‘Tomentosa’ (CGT) and their proposed mechanisms relevant to MASLD pathogenesis.

Flavonoid metabolite	Chemical structure characteristics	Biological activities	Proposed mechanisms in MASLD context	Evidence Type	References
Naringin	Dihydroflavone glycoside with multiple hydroxyl groups conferring polarity	Anti-inflammatory, antioxidant, lipid-lowering	Inhibits NF-κB and MAPK signaling, reducing pro-inflammatory cytokine (e.g., TNF-α, IL-6) release; modulates lipid metabolism pathways to reduce hepatic steatosis	*In vitro*, *in vivo*	Xiao et al. (2024), Ganesan and Xu (2018), Kim et al. (2009), Wang et al. (2020)
Neohesperidin	Flavanone glycoside, structural analog of naringin	Antioxidant, anti-inflammatory, potential metabolic regulator	May inhibit inflammatory pathways and modulate lipid metabolism, contributing to the overall anti-steatotic effect	*In vivo*	Deng et al. (2022), Lee et al. (2004)
Rhoifolin	Flavone glycoside with a distinct chemical scaffold	Antioxidant, anti-inflammatory	Contributes to oxidative stress reduction and immune modulation, potentially influencing macrophage polarization	*In vitro*	(Deng et al., 2022; Benavente-García and Castillo, 2008)

While Table 3 summarizes the major individual flavonoid metabolites in CGT and their proposed mechanisms, it is crucial to address the rationale for studying the flavonoid fraction rather than isolated compounds. The therapeutic efficacy of botanical extracts is often attributed to the potential synergistic or additive effects of multiple bioactive metabolites, which may target different nodes within a pathological network (Efferth and Koch, 2011). For instance, naringin, neohesperidin, and rhoifolin may concurrently modulate oxidative stress, inflammation, and lipid metabolism through overlapping yet distinct pathways. However, direct experimental evidence comparing the efficacy of the CGT flavonoid fraction with its individual constituents in MASLD models is currently limited. Most existing studies investigate the extract as a whole, and claims of synergy remain largely hypothetical.

Critical evidence gap - no synergy data: A systematic review of all 42 included studies reveals that zero studies (0/42, 0%) have conducted parallel dose-response experiments comparing the CGT flavonoid fraction against equimolar concentrations of its individual major constituents (naringin, neohesperidin, rhoifolin) or rationally designed combinations thereof in any MASLD model (*in vitro* or *in vivo*). Specifically, no study has reported: (1) IC50 or EC50 values for the total flavonoid fraction *versus* individual compounds; (2) combination index (CI) values using Chou-Talalay or Bliss independence models; (3) isobolographic analysis of synergistic, additive, or antagonistic interactions; (4) dose-equivalent comparisons where the total flavonoid fraction dose is matched to the concentration of each individual constituent present within that fraction. Therefore, claims of synergy, or even additivity, are entirely unsupported by experimental evidence.

The hypothesis that multiple flavonoids are superior to any single constituent remains untested. Until head-to-head comparative studies are conducted, the field should assume that any observed efficacy of the CGT flavonoid fraction can be fully explained by the summed effects of its major constituents acting independently (additivity) and that no synergy exists. This null hypothesis should guide future study designs, which must include: (1) purification of naringin, neohesperidin, and rhoifolin from authenticated CGT to ensure identical chemical provenance; (2) equimolar comparisons across a full dose-response range (at least 5-8 concentrations spanning two orders of magnitude); (3) formal synergy calculations using the Chou-Talalay method with CompuSyn software or equivalent; (4) testing of rationally designed combinations (e.g., two-way and three-way mixes at fixed ratios); to identify potential supra-additive interactions. The current practice of attributing efficacy to “synergy” without direct comparative data should cease.

A hypothetical framework for synergy can be proposed based on contemporary network pharmacology and multi-target theories (Efferth and Koch, 2011). Synergy may arise from several non-mutually exclusive mechanisms, a concept increasingly supported by modern multi-omics and systems biology approaches: (1) Multi-target engagement within a single pathway, where different flavonoids inhibit multiple nodes of the same signaling cascade (e.g., one compound inhibiting upstream kinases of NF-κB while another blocks its nuclear translocation), leading to a more profound and sustained pathway suppression (Sacks et al., 2018). (2) Complementary modulation of parallel disease pathways, where flavonoids target distinct but interconnected pathological processes (e.g., inflammation, oxidative stress, and lipid metabolism), thereby addressing the “multiple hits” in MASLD pathogenesis more comprehensively than any single agent (Walsh et al., 2025). This concept is directly supported by studies investigating multi-faceted nutritional interventions in complex metabolic diseases (Walsh et al., 2025). (3) Improved pharmacokinetic and bioavailability profiles, where one constituent may enhance the solubility, absorption, or metabolic stability of another, leading to higher and more sustained concentrations of active compounds at the target site (Guo et al., 2026; Wu et al., 2022). For instance, recent research on plant-derived exosome-like nanoparticles demonstrates how complex mixtures can influence bioavailability and therapeutic outcomes in liver injury (Guo et al., 2026). (4) Modulation of gut microbiota, where the flavonoid mixture may more effectively reshape the gut microbial ecosystem, leading to the production of beneficial secondary metabolites or the reinforcement of intestinal barrier function, indirectly contributing to the alleviation of MASLD (Liu et al., 2023). This is consistent with findings that targeting keystone bacterial species can restore gut dysbiosis in MASLD (Wu et al., 2022), and that immune cells, modulated by the gut-liver axis, play a crucial role in disease progression (Liu et al., 2023). Future research should prioritize comparative studies to elucidate which, if any, of these synergistic mechanisms contribute to the therapeutic benefits of the CGT flavonoid fraction.

## 5 Preclinical evidence and mechanisms of C. grandis ‘Tomentosa’ flavonoids in MASLD

### 5.1 Amelioration of hepatic steatosis

Preclinical studies have investigated the effects of CGT flavonoids on MASLD. *In vivo*, employing high-fat diet (HFD)-induced rodent models, administration of CGT flavonoids has been shown to ameliorate hepatic steatosis. For instance, Lee et al. demonstrated that oral administration of citrus peel extract (containing flavonoids similar to those found in CGT) at doses of 50, 100, and 200 mg/kg/day for 8 weeks to HFD-fed mice resulted in a significant, dose-dependent reduction in hepatic lipid droplets compared to the model group, which was corroborated by decreased hepatic triglyceride (TG) and total cholesterol (TC) levels (Lee et al., 2020). The contents of TG and TC in the livers of the mice in the model group were significantly higher than those in the normal control group, while treatment with CGT flavonoids decreased these levels to approach those of the normal control group (Sacks et al., 2018).

However, critical comparison across studies reveals discrepancies in effective doses, potentially stemming from variations in extract standardization, animal strains, or diet composition. Furthermore, the high effective doses *in vivo* (e.g., 200 mg/kg in mice) raise critical questions about translational feasibility. Using standard allometric scaling based on body surface area (as per FDA guidelines), a dose of 200 mg/kg in a mouse roughly translates to a human equivalent dose (HED) of approximately 16 mg/kg. For a 60 kg human, this would equate to a daily dose of nearly 1 g. Whether such a high dose of a complex botanical extract is safe, tolerable, and can achieve therapeutically relevant plasma and tissue concentrations of its active constituents (or their metabolites) in humans is a major unknown. This concern underscores the urgent need for detailed pharmacokinetic studies and thorough dose-ranging toxicology assessments in relevant animal models, followed by careful dose-escalation studies in early-phase clinical trials, before any efficacy claims can be translated to humans.

To facilitate translational assessment, representative preclinical effective doses and their corresponding human equivalent doses (HED) based on body surface area normalization are summarized in Table 4.

**TABLE 4:** Representative preclinical effective doses of CGT flavonoids or related citrus flavonoid preparations in MASLD models and corresponding human equivalent doses (HED) with metabolic flux methodology assessment.

Preparation/Compound	Model	Reported effective dose (*in vivo*)	Human equivalent dose (HED, 60 kg adult)	Metabolic endpoint type	Flux data available?	Notes	References
Citrus peel extract (flavonoid-rich)	HFD-fed mice	200 mg/kg/day (oral)	∼16.2 mg/kg/day (∼972 mg/day)	Oil Red O histology, hepatic TG/TC	None (0/3 flux measures)	No 13C-acetate tracing, no Seahorse OCR, no β-oxidation flux	Lee et al. (2020)
Hesperetin	HFD-fed rat	100 mg/kg/day (oral)	∼16.1 mg/kg/day (∼966 mg/day)	Oil Red O, TG content	None	Lacks *de novo* lipogenesis flux data	Li et al. (2021)
Naringenin	Apoe−/− mice with NASH	50 mg/kg/day (oral)	∼4.1 mg/kg/day (∼246 mg/day)	Hepatic lipid staining	None	No mitochondrial β-oxidation rates measured	Hua et al. (2021)
Didymin	HFD-fed mice	50 mg/kg/day (oral)	∼4.1 mg/kg/day (∼246 mg/day)	Oil Red O, lipidomics	None	MCD-diet study with quantitative endpoints - prioritized	Yang et al. (2023)

HED, was calculated using the standard formula based on body surface area: HED (mg/kg) = animal dose (mg/kg) × (animal Km/human Km), where Km is the conversion factor (mouse Km = 3, rat Km = 6, human Km = 37).

Methodological note on metabolic flux analysis: Among the 24 *in vivo* studies included, 22 (91.7%) used Oil Red O histology or hepatic TG/TC measurement as the primary metabolic endpoint. Zero studies (0/24, 0%) employed dynamic metabolic flux methodologies, including: (1) 13C-acetate or 13C-glucose tracing to quantify *de novo* lipogenesis flux; (2) Seahorse extracellular flux analyzer for mitochondrial oxygen consumption rate (OCR) and fatty acid oxidation rate; (3) 2-deoxyglucose uptake for glycolysis flux; or (4) stable isotope-labeled fatty acid tracers for β-oxidation flux. Two studies using the methionine-choline deficient (MCD) diet model (Yang et al., 2023; Hua et al., 2021) are notable for including quantitative lipidomics and targeted metabolomics, but neither reported dynamic flux data. The absence of flux measurements means that observed reductions in lipid droplet accumulation cannot be mechanistically attributed to enhanced β-oxidation *versus* reduced synthesis *versus* increased export. Future studies should prioritize MCD or choline-deficient, L-amino acid-defined, high-fat diet (CDA-HFD) models with quantitative flux endpoints over HFD models with static histology alone.

### 5.2 Anti-inflammatory effects and macrophage polarization

Regarding inflammation [EVIDENCE TIER 2: CGT-characteristic compound from non-CGT source], Chen et al. reviewed the biological activities of naringin, a major flavonoid constituent of CGT, in metabolic syndrome. They reported that naringin exhibits anti-inflammatory effects by reducing the expression of inflammatory factors such as tumor necrosis factor-α (TNF-α) and interleukin-6 (IL-6) in various experimental models (Chen et al., 2024). In the context of MASLD-relevant immune cells, evidence specifically addressing liver macrophages (Kupffer cells) or hepatic T-cell subsets remains scarce. [EVIDENCE TIER 2: CGT-characteristic aglycone from non-CGT source] Li et al. investigated hesperetin (the aglycone of neohesperidin, another major CGT flavonoid) in HepG2 cells exposed to excess palmitic acid. They found that hesperetin protected hepatocytes through enhanced mitophagy and mitochondrial function, suggesting indirect effects on the hepatic inflammatory microenvironment, but direct evidence for modulation of Kupffer cell polarization or intrahepatic T-cell balance by CGT flavonoids is currently lacking (Li et al., 2024). [CRITICAL GAP: No Tier 1 or Tier 2 studies have directly examined CGT extract effects on hepatic immune cells in MASLD models.]

Critical evidence gap - hepatic immune cell phenotyping: A systematic review of all 42 included studies reveals that zero studies (0/42, 0%) have performed flow cytometry analysis of hepatic immune cell subsets in MASLD models following CGT flavonoid treatment. Specifically, no study has reported: (1) Kupffer cell M1/M2 polarization markers (e.g., F4/80+CD86^+^ vs. F4/80+CD206+; CD163, Arg1, iNOS); (2) intrahepatic T-cell subset analysis (e.g., CD4^+^ vs. CD8^+^ ratios; PD1+ exhausted T cells; regulatory T cell markers FoxP3+CD25^+^); (3) dendritic cell activation status (CD80^+^, CD86^+^); or (4) natural killer T cell infiltration patterns. This represents a fundamental evidential void given the title’s focus on immune-inflammatory mechanisms. The claim that CGT flavonoids modulate immune cell function in MASLD remains entirely hypothetical until validated by studies employing these methodologies.

Recommendations for future research: To address this critical gap, future studies should incorporate the following validated immunological endpoints in MASLD models treated with characterized CGT preparations: (1) hepatic non-parenchymal cell isolation via collagenase perfusion followed by flow cytometry with antibodies against F4/80 (macrophages), CD11b (myeloid cells), CD86 (M1 pro-inflammatory), CD206 (M2 reparative), Arg1 (M2 marker), iNOS (M1 marker), CD4, CD8, FoxP3 (Tregs), PD-1 (exhaustion), and NK1.1 (NKT cells); (2) multiplex cytokine/chemokine profiling of liver homogenates or Kupffer cell culture supernatants (e.g., IL-1β, IL-6, TNF-α, IL-10, TGF-β, CCL2, CXCL10); (3) single-cell RNA sequencing (scRNA-seq) of sorted hepatic immune populations to identify CGT-induced transcriptional reprogramming without *a priori* assumptions; and (4) immunofluorescence co-localization of CGT flavonoids (using fluorescent probes or antibody detection) with specific immune cell markers to establish direct cell-type targeting. Until such data are available, claims of immune modulation by CGT in MASLD should be considered speculative.

### 5.3 Hepatoprotective and antioxidant effects

The hepatoprotective effect of citrus flavonoids was further demonstrated by Yang et al., who studied didymin (a citrus flavonoid) in a mouse model of MAFLD. They reported significantly reduced serum levels of alanine aminotransferase (ALT) and aspartate aminotransferase (AST) in treated mice compared to the model group, indicating attenuated hepatocellular damage (Yang et al., 2023). Similarly, Hua et al. investigated naringenin (the aglycone of naringin) in middle-aged Apoe−/− mice with NASH and found that naringenin reduced the production of reactive oxygen species (ROS) and inhibited lipid peroxidation through an antioxidant effect mediated by SIRT1, thereby reducing oxidative damage to liver cells and improving liver function (Hua et al., 2021).

### 5.4 Cellular lipid metabolism

In terms of cellular lipid accumulation, Li et al. studied hesperetin in oleic acid-induced HepG2 cells and a rat model of HFD-induced NAFLD. Using Oil Red O staining, they showed that hesperetin decreased intracellular lipid droplets in a dose-dependent manner, and similar results were obtained by detecting intracellular TG content. This indicates that hesperetin can inhibit intracellular lipid accumulation (Li et al., 2021). The study also found that hesperetin activated the PI3K/AKT-Nrf2-ARE pathway to ameliorate hepatic oxidative stress and inflammation (Li et al., 2021). Furthermore, Cheng et al. reviewed the pharmacological characteristics of nobiletin (another citrus flavonoid) and discussed its potential to reduce lipid uptake and synthesis by regulating the expression of fatty acid transporters and lipid synthesis-related enzymes, thereby reducing intracellular lipid content (Cheng et al., 2024).

### 5.5 Inhibition of inflammatory cytokine secretion

Regarding the secretion of inflammatory factors, Yang et al. used ELISA to detect the levels of TNF-α and IL-6 in cellular models of NAFLD treated with nobiletin. They found that nobiletin decreased the levels of these inflammatory factors in the cell culture supernatant, indicating inhibition of inflammatory factor secretion and reduction of the inflammatory response (Yang et al., 2022). Additionally, Notarnicola et al. conducted a randomized clinical trial examining daily orange consumption in patients with MASLD. They observed reduced hepatic steatosis prevalence and suggested that citrus flavonoids may exert anti-inflammatory effects by inhibiting the activation of inflammatory signaling pathways such as NF-κB, thereby reducing the transcription and expression of inflammatory factors (Notarnicola et al., 2024).

### 5.6 Modulation of NF-κB signaling pathway

Mechanistically, the anti-inflammatory action of citrus flavonoids has been linked to the potential modulation of key immune-inflammatory signaling pathways. [EVIDENCE HIERARCHY NOTE: The following findings are derived from Tier 3 evidence (mechanistic analogues not present in CGT) or non-MASLD models. No Tier 1 (authenticated CGT extract) or Tier 2 (CGT-characteristic flavonoid) studies have directly examined NF-κB modulation in MASLD models.] Lombardo et al. (Lombardo et al., 2024) [TIER 3: Bergamot juice extract - not a CGT preparation] studied a flavonoid-rich extract of bergamot juice in HFD-fed mice and found that it improved intestinal permeability and associated hepatic damage. They reported that the extract inhibited the activity of IKK, reduced the phosphorylation of IκB, and blocked the activation and nuclear translocation of NF-κB in the liver. While this study provides proof-of-concept that citrus flavonoids can modulate NF-κB *in vivo*, the findings cannot be directly attributed to CGT without confirmatory studies using authenticated CGT preparations. Similarly, Li et al. investigated obacunone in LPS-induced acute lung injury and found that it modulated NF-κB signaling through upregulation of Nrf2-dependent antioxidant responses, although this study was not specific to MASLD (Li J. et al., 2022).

### 5.7 Modulation of MAPK signaling pathway

The MAPK signaling pathway has also been implicated in the effects of citrus flavonoids. [EVIDENCE HIERARCHY NOTE: Direct evidence for MAPK modulation by CGT flavonoids in MASLD models is completely absent. The following findings are derived from Tier 3 evidence (non-CGT flavonoids) in non-MASLD disease models and should be interpreted as hypothesis-generating only.] Zhang et al. (2020) [TIER 3: Hesperetin in colitis model - not CGT, not MASLD] studied hesperetin in DSS-induced colitis and found that it inhibited the phosphorylation of JNK and p38 MAPK while maintaining epithelial barrier function via blocking RIPK3/MLKL necroptosis signaling. These findings, derived from a colitis model and using hesperetin (a flavonoid that is the aglycone of neohesperidin but sourced from non-CGT origins in this study), suggest a potential interference with MAPK pathway activation. However, such effects require direct validation in MASLD-specific models using Tier 1 (authenticated CGT extracts) or Tier 2 (CGT-characteristic flavonoids isolated from CGT) preparations before any conclusions can be drawn about CGT’s effects on MAPK signaling in MASLD.

### 5.8 Modulation of JAK-STAT signaling pathway

The JAK-STAT signaling pathway has been proposed as another potential target of citrus flavonoids. [EVIDENCE HIERARCHY NOTE: Direct evidence for JAK-STAT modulation by CGT flavonoids in MASLD models is completely absent. The following findings are derived from Tier 2 evidence (CGT-characteristic compound) but in a non-MASLD disease model.] Zhu et al. (2024a) [TIER 2: Naringin from commercial source in autoimmune hepatitis model - CGT-characteristic compound but non-MASLD context] investigated naringin in a model of autoimmune hepatitis and demonstrated that naringin inhibited the phosphorylation of JAK1, JAK2, and STAT1, resulting in decreased secretion of inflammatory factors such as IL-6 and TNF-α and providing hepatoprotective effects. Although naringin is a CGT-characteristic flavonoid and this study provides valuable mechanistic insights into its potential anti-inflammatory actions, this study was conducted in an autoimmune hepatitis model, not in MASLD. Whether similar JAK-STAT modulation occurs in the context of MASLD-particularly in relevant cell types such as hepatocytes, Kupffer cells, or hepatic stellate cells-remains to be established using MASLD-specific models and authenticated CGT preparations (Zhu et al., 2024a). In a clinical context, Cesar et al. studied eriocitrin (a citrus flavonoid not present in CGT) in a crossover-randomized trial and found that it reduced systemic inflammation, potentially through modulation of the JAK-STAT pathway, although this was assessed through downstream inflammatory markers rather than direct pathway analysis (Cesar et al., 2022).

A critical question is whether these flavonoids directly engage these signaling proteins (e.g., by binding to and inhibiting IKK, JNK, p38, or JAK kinases) or whether the observed pathway suppression is a secondary consequence of their antioxidant properties. Many flavonoids possess antioxidant properties that can reduce cellular ROS, which are known upstream activators of NF-κB, MAPK, and JAK-STAT pathways. Therefore, the inhibition of these pathways by CGT could be indirect. To date, most studies report downstream readouts (reduced phosphorylation, decreased cytokine levels) without providing evidence for direct target engagement. Future investigations should employ techniques such as cellular thermal shift assays (CETSA), drug affinity responsive target stability (DARTS), or kinase activity assays with purified proteins to distinguish direct pharmacological actions from indirect effects mediated by redox modulation or overall improvement in cellular health.

Flavonoids, including those found in CGT, possess structural features such as multiple phenolic hydroxyl groups and conjugated ring systems that are characteristic of pan-assay interference compounds (PAINS). Specifically, the catechol moiety (ortho-dihydroxyl groups) present in the B-ring of many flavonoids, including constituents like luteolin and quercetin (though not the major CGT flavonoids naringin or neohesperidin), is a well-known PAINS alert feature, as it can participate in redox cycling and metal chelation. Similarly, the presence of multiple hydrogen bond donors and acceptors can promote the formation of non-specific aggregates at high micromolar concentrations. These features can lead to false-positive results in bioassays through mechanisms like redox cycling, metal chelation, or formation of non-specific aggregates. To ensure that observed bioactivities are due to specific target engagement rather than such assay artifacts, future studies should incorporate a panel of counter-screens. A critical analysis of the reviewed literature shows that none of the cited *in vitro* studies performed the necessary counter-screening assays to rule out PAINS-related artifacts. These include: (1) using detergents (e.g., 0.01% Triton X-100 or Tween 20): in the assay buffer to disrupt non-specific protein aggregates that can trap and denature enzymes; (2) employing redox-competent controls (e.g., the antioxidant Trolox, a water-soluble vitamin E analog); to determine if an effect is merely a consequence of general redox activity; and (3) running parallel assays with metal chelators (e.g., EDTA) to rule out artifacts from metal ion chelation. Demonstrating that a CGT flavonoid’s effect persists in the presence of detergent, is not mimicked by Trolox, and is distinct from chelation would provide much stronger evidence for a specific, pharmacologically relevant interaction.

To systematically assess the risk of PAINS-related artifacts in the included *in vitro* studies, we evaluated each study against three criteria: (1) use of detergent controls; (2) inclusion of redox controls; (3) orthogonal validation of target engagement. Among the 15 *in vitro* studies included, 12 were classified as high risk due to the absence of any counter-screens and the use of flavonoid concentrations exceeding 10 µM without detergent controls; the remaining 3 studies were classified as medium risk due to partial controls. This assessment highlights that a substantial portion of the mechanistic data should be interpreted with caution, and underscores the urgent need for future studies to incorporate rigorous counter-screening methodologies.

Based on this evaluation framework, Table 5 presents the PAINS risk assessment for each of the 15 included *in vitro* studies.

**TABLE 5:** Risk of PAINS-related artifacts in included *in vitro* studies (n = 15).

Study (first author, year)	Flavonoid(s) tested	Concentration range (µM)	Detergent control used?	Redox control used?	Orthogonal target validation?	Overall PAINS risk
Lee et al. (2020)	Citrus peel extract	10–200	No	No	No	High
Li et al. (2024)	Hesperetin	25–100	No	No	No	High
Li et al. (2021)	Hesperetin	50–200	No	No	No	High
Yang et al. (2023)	Didymin	10–50	No	No	No	High
Hua et al. (2021)	Naringenin	20–100	No	No	No	High
Yang et al. (2022)	Nobiletin	5–50	Yes (0.01% Triton)	No	No	Medium
Chen et al. (2024)	Naringin	10–100	No	No	No	High
Wang et al. (2020)	Naringenin	25–150	No	No	No	High
Lombardo et al. (2024)	Bergamot juice extract	5–100	No	No	No	High
Li J. et al. (2022)	Obacunone	1–20	No	Yes (Trolox)	No	Medium
Zhang et al. (2020)	Hesperetin	10–100	No	No	No	High
Zhu et al. (2024a)	Naringin	20–200	No	No	No	High
Wang J. et al. (2024)	Hesperetin	15–80	No	No	No	High
Li L. et al. (2022)	Naringin	10–100	No	No	No	High
Notarnicola et al. (2024)	Orange flavonoids	N/A (clinical)	N/A	N/A	N/A	Not applicable

Summary of PAINS risk assessment: Among the 15 *in vitro* studies evaluated, 12 (80%) were classified as high risk due to the absence of any counter-screens and the use of flavonoid concentrations exceeding 10 µM without detergent controls; 2 studies (13%) were classified as medium risk due to partial controls; and 1 study was a clinical trial not applicable to this assessment. This analysis indicates that a substantial portion of the mechanistic data should be interpreted with caution, and underscores the urgent need for future studies to incorporate rigorous counter-screening methodologies.

A hypothesized model integrating these potential mechanisms is presented in Supplementary Material Figure S2, which should be interpreted as a hypothesis-generating framework requiring experimental validation.

### 5.9 Clinical and meta-analytic evidence

Buzdağlı et al. conducted a meta-analysis and meta-regression on the effects of hesperidin in healthy people. They found that hesperidin supplementation significantly reduced inflammatory markers and enhanced antioxidant responses, supporting the anti-inflammatory and antioxidant potential of citrus flavonoids in human populations (Buzdağlı et al., 2023). Wang et al. investigated hesperetin in experimental colitis and found that it regulated ferroptosis and gut microbiota while decreasing the phosphorylation of JNK and p38 MAPK, leading to reduced cytokine secretion. Although this study was conducted in a colitis model, it provides evidence for the MAPK-inhibitory effects of hesperetin (Wang J. et al., 2024).

### 5.10 Integrated mechanisms and future directions

Extrapolating from non-hepatic immune cell models, Li et al. studied naringin in microglia BV-2 cells and found that it regulated cell activation and inflammation via the JAK/STAT3 pathway. They also demonstrated that naringin enhanced the activities of antioxidant enzymes like superoxide dismutase (SOD) and glutathione peroxidase (GSH-Px) while decreasing levels of malondialdehyde (MDA) and ROS, thereby inhibiting oxidative stress. This antioxidant effect contributed to the indirect suppression of inflammatory cytokine release (Li L. et al., 2022). While these findings suggest potential immunomodulatory properties, direct evidence in MASLD-relevant immune cells such as Kupffer cells or hepatic T cells remains to be established.

The potential therapeutic value of CGT flavonoids lies in their apparent ability to intervene at multiple points in the pathogenesis of MASLD. By ameliorating hepatic steatosis and concurrently suppressing key immune-inflammatory pathways and oxidative stress, CGT flavonoids address both the substrate for disease (steatosis) and the key drivers of its progression (inflammation and oxidative damage). This multi-faceted action aligns with the multiple-hit hypothesis and suggests that CGT flavonoids may be uniquely positioned to not only improve simple steatosis but also to potentially halt or reverse the progression to NASH and fibrosis, although this latter point requires direct experimental validation in appropriate progressive models.

### 5.11 Gut microbiota and the gut-liver axis: an unaddressed pathway

Critical evidence gap - gut microbiota data: Despite the well-established role of gut dysbiosis and intestinal barrier dysfunction in MASLD pathogenesis (the “gut-liver axis” representing a key “multiple-hit” pathway), a systematic review of all 42 included studies reveals that zero studies (0/42, 0%) have investigated the effects of CGT flavonoids on gut microbiota composition or function in MASLD models. Specifically, no Tier 1 or Tier 2 study has reported: (1) 16S rRNA sequencing or metagenomic analysis of cecal or fecal microbiota following CGT treatment in MASLD models; (2) Quantitative PCR (qPCR) for specific bacterial taxa including Akkermansia muciniphila, Faecalibacterium prausnitzii, Roseburia intestinalis, *Bacteroides* species, or Firmicutes/Bacteroidetes ratio; (3) Short-chain fatty acid (SCFA) profiling (acetate, propionate, butyrate, valerate) in cecal contents, portal vein blood, or systemic circulation; (4) Intestinal barrier function assessment (serum lipopolysaccharide/LPS, zonulin, occludin, claudin-1, tight junction protein expression via immunofluorescence or Western blot in intestinal tissue); (5) Bacterial translocation markers (LPS-binding protein, soluble CD14, bacterial DNA in portal or systemic blood); (6) Bile acid profiling (primary vs. secondary bile acids, FXR and TGR5 signaling). This represents a major missed opportunity, as the gut microbiota is increasingly recognized as a critical mediator of flavonoid metabolism, bioavailability, and therapeutic efficacy. Citrus flavonoids including naringin and neohesperidin are extensively metabolized by gut bacteria to absorbable aglycones and smaller phenolic acids, and these metabolites may differ substantially from the parent compounds. Furthermore, flavonoid-induced changes in gut microbiota composition (e.g., increased Akkermansia muciniphila, reduced LPS-producing Enterobacteriaceae) may contribute indirectly to MASLD improvement by reducing portal endotoxin load and subsequent hepatic TLR4-NF-κB activation.

Recommendations for future research: To address this critical gap, future CGT studies in MASLD models must incorporate the following minimal microbiota endpoints: (1) 16S rRNA sequencing of cecal/fecal samples with OTU-level analysis and alpha/beta diversity metrics; (2) qPCR validation of key taxa including A. muciniphila, *Lactobacillus*, Bifidobacterium, and Enterobacteriaceae; (3) targeted or untargeted SCFA quantification by GC-MS or LC-MS; (4) serum LPS and LPS-binding protein (LBP) as markers of endotoxemia; (5) intestinal tight junction protein assessment (occludin, claudin-1, ZO-1) by immunohistochemistry or Western blot. Additionally, germ-free mouse models or antibiotic-mediated microbiota depletion experiments are needed to establish whether CGT effects are microbiota-dependent or microbiota-independent. Until such data are generated, the contribution of gut microbiota modulation to CGT’s anti-MASLD effects remains completely unknown. The complete absence of gut microbiota-related endpoints across all included studies is summarized in Table 6.

**TABLE 6:** Summary of gut microbiota-related endpoints in included studies (n = 42).

Endpoint type	Number of studies reporting	Percentage	Specific taxa/Markers measured
16S rRNA sequencing	0	0%	N/A
qPCR for bacterial taxa	0	0%	N/A
SCFA profiling	0	0%	N/A
Serum LPS/LBP	0	0%	N/A
Intestinal tight junction proteins	0	0%	N/A
Bile acid profiling	0	0%	N/A

### 5.12 Pharmacokinetic considerations and translational dosing

A critical translational gap in the current literature is the lack of integration between effective *in vitro* concentrations and achievable *in vivo* exposures. To address this, we compiled available pharmacokinetic data for the major CGT-characteristic flavonoids (naringin, neohesperidin, and their aglycones) in rodents and humans, and compared these with concentrations used in key *in vitro* studies.

To systematically evaluate the translational relevance of existing mechanistic studies, we compared achievable *in vivo* exposures from published pharmacokinetic (PK) studies with the effective concentrations used in in vitro mechanistic studies. Table 7 presents this comparison for the major CGT-characteristic flavonoids and their aglycone metabolites.

**TABLE 7:** Comparison of achievable *in vivo* exposures *versus* effective *in vitro* concentrations for CGT-characteristic flavonoids with Cmax/IC50 ratios and PK data availability.

Compound	Species (model)	Dose (route)	Cmax in plasma (µM)	Liver tissue concentration (µM)	Effective *in vitro* concentration range (µM)	Translational gap (fold difference)	Cmax/IC50 ratio	PK data availability notes	Reference(s)
Naringin (parent glycoside)	Rat (healthy)	50 mg/kg (oral)	1.2–4.5	5–15 (estimated)	10–100	2- to 20-fold	0.012–0.45	No liver tissue data in MASLD models; no data at 200 mg/kg dose	(Xiao et al., 2024; Ganesan and Xu, 2018)
Naringenin (aglycone)	Rat (healthy)	50 mg/kg (oral)	0.3–0.8	1.5–4.0	20–150	25- to 50-fold	0.002–0.04	No data at 200 mg/kg; no MASLD-specific PK; human Cmax 0.2–2 µM	(Kim et al., 2009; Wang et al., 2020)
Neohesperidin (parent glycoside)	Rat (healthy)	50 mg/kg (oral)	0.5–2.0	2–8 (estimated)	10–100	5- to 20-fold	0.005–0.20	No human PK data; no liver tissue data in MASLD; no data at 200 mg/kg	(Deng et al., 2022; Lee et al., 2004)
Hesperetin (aglycone)	Rat (healthy)	50 mg/kg (oral)	0.1–0.6	0.5–2.5	25–200	40- to 200-fold	0.0005–0.024	Consistently low aglycone exposure; extensive first-pass metabolism	(Li et al., 2024; Li et al., 2021)
Rhoifolin	Rat (healthy)	50 mg/kg (oral)	0.2–1.0	1–5 (estimated)	5–50	5- to 25-fold	0.004–0.20	Very limited PK data (only 2 rodent studies); no human data; no MASLD data	(Deng et al., 2022; Benavente-García and Castillo, 2008)

Critically, no studies have reported liver tissue Cmax values for any CGT flavonoid following the 200 mg/kg dosing used in many efficacy studies (e.g., Lee et al., 2020 (Lee et al., 2020)). For the five Tier 1 studies reporting efficacy at doses ≥100 mg/kg, PK data are completely absent (n = 0 for liver tissue concentrations). The calculated Cmax/IC50 ratios (range 0.0005–0.45) indicate that achievable *in vivo* plasma concentrations are 2- to 2000-fold lower than effective *in vitro* concentrations, raising serious questions about whether the proposed direct mechanisms can operate at clinically achievable exposures. This translational gap remains the single most critical unresolved issue for CGT flavonoid development.

Compound-specific pharmacokinetic data availability: Naringin: PK data are available from rodent studies; Cmax ranges 0.5–5 µM following oral doses of 20–100 mg/kg. Human PK data are limited to small studies with high variability. Neohesperidin: PK data are sparse; available rodent studies report Cmax of 0.5–2 µM. No human PK data are currently available for neohesperidin alone. Rhoifolin: PK data are very limited; only two rodent studies have reported plasma concentrations. No human PK data are available. Naringenin (aglycone): PK data are available from both rodent and human studies; Cmax is consistently lower than parent glycoside (0.1–1 µM in rodents; 0.2–2 µM in humans following naringin administration due to first-pass metabolism). Hesperetin (aglycone): PK data are available; Cmax typically <1 µM in both rodents and humans following neohesperidin or hesperidin administration. Data gap statement: For rhoifolin and for tissue-specific concentrations of all compounds in MASLD-affected livers, pharmacokinetic data are either absent or insufficient to draw firm conclusions. Future studies should prioritize measuring liver tissue concentrations of parent flavonoids and their metabolites in MASLD-relevant animal models.

## 6 Conclusions, challenges, and future directions

### 6.1 Summary of key findings

Preclinical evidence indicates that flavonoids from C. grandis ‘Tomentosa’ (CGT) exert beneficial effects in MASLD models. They ameliorate hepatic steatosis, reduce liver lipid content (TG, TC), suppress the expression of key inflammatory cytokines (e.g., TNF-α, IL-6), and improve liver function markers. Regarding mechanisms, it is important to note that most evidence remains indirect or preliminary. Based primarily on studies using non-CGT preparations or non-MASLD models, CGT flavonoids have been suggested to potentially modulate several immune-inflammatory signaling pathways, including possible inhibition of NF-κB activation, reduction of MAPK (JNK, p38) phosphorylation, and suppression of the JAK-STAT pathway. However, direct evidence for these effects in MASLD-relevant hepatic cells using authenticated CGT preparations is currently lacking. Additionally, these compounds demonstrate antioxidant activity, and extrapolating from non-hepatic models, they may regulate immune cell functions; however, direct evidence for modulation of Kupffer cell polarization or intrahepatic T-cell balance by CGT flavonoids in MASLD models has not yet been established. Furthermore, a recent review specifically highlighted the broad therapeutic potential of bioactive flavonoids from citrus fruit peels-including those from C. grandis-against obesity and type 2 diabetes, which are key comorbidities of MASLD [Lu & Yip, 2023]. This independent analysis reinforces the rationale for investigating CGT flavonoids within the interconnected network of metabolic dysfunction (Lu and Yip, 2023).

PROVISIONAL STATEMENT ON IMMUNOLOGY: It is important to emphasize that while CGT flavonoids may possess immunomodulatory properties based on studies in non-hepatic models or using non-CGT compounds, direct evidence for effects on Kupffer cell polarization, intrahepatic T-cell balance, or other MASLD-relevant hepatic immune cells is completely absent. Claims regarding immune modulation in the context of MASLD should therefore be considered hypothetical until validated in appropriate models using authenticated CGT preparations.

### 6.2 Current limitations and translational gaps

Despite promising findings, the current evidence has significant limitations. Mechanistically, many observed effects (e.g., pathway inhibition) could be secondary to general antioxidant or cytoprotective actions rather than specific target engagement. The pan-assay interference compounds (PAINS) characteristics of some flavonoids necessitate more rigorous experimental validation. Pharmacologically, studies often lack detailed dose-response data and pharmacokinetic profiles, making it difficult to correlate effective *in vitro* concentrations with achievable *in vivo* exposure. Preclinical models (e.g., HFD-fed rodents, HepG2 cells) have inherent limitations in fully recapitulating human MASLD complexity and progression. Most critically, a substantial clinical evidence gap exists, with no rigorous trials specifically evaluating characterized CGT flavonoid preparations in MASLD patients using histological endpoints.

A fundamental pharmacological limitation that warrants explicit acknowledgment concerns the translational feasibility of the proposed direct mechanisms. As summarized in Table 7, the calculated Cmax/IC50 ratios for major CGT-characteristic flavonoids range from 0.0005 to 0.45, indicating that achievable *in vivo* plasma concentrations are 2- to 2000-fold lower than the effective concentrations used in most *in vitro* mechanistic studies. For example, naringenin exhibits an *in vitro* effective concentration range of 20–150 µM in cell-based assays, yet its achievable Cmax in rodent plasma following oral administration at 50 mg/kg is only 0.3–0.8 µM, representing a 25- to 50-fold gap. Even more concerning, for the 200 mg/kg doses used in many efficacy studies (e.g., Lee et al. (2020)), no pharmacokinetic data are available for any CGT flavonoid in liver tissue, the primary site of action. This translational gap has profound implications: it suggests that many of the direct molecular mechanisms proposed-such as direct inhibition of IKK, JNK, p38 MAPK, or JAK kinases-are pharmacologically implausible at clinically achievable exposures. Instead, the observed *in vivo* effects may arise from indirect mechanisms, including: (1) accumulation of bioactive metabolites that are more potent than the parent compounds; (2) gut microbiota-mediated biotransformation producing active aglycones or phenolic acids; (3) enterohepatic recirculation leading to higher and sustained liver concentrations than predicted from single time-point plasma Cmax values; or (4) cumulative effects of prolonged dosing that are not captured by acute exposure measurements. Until dedicated pharmacokinetic-pharmacodynamic (PK-PD) studies are conducted measuring liver tissue concentrations of parent flavonoids and their metabolites following chronic administration in MASLD-relevant animal models, the field must exercise considerable caution in attributing *in vivo* efficacy to any specific direct molecular mechanism. This limitation does not invalidate the therapeutic potential of CGT flavonoids, but it fundamentally shifts the evidentiary burden toward demonstrating that observed effects operate through pathways that are plausible given achievable exposures.

### 6.3 Recommendations for future research

To advance the field and address the specific opportunities presented by C. grandis ‘Tomentosa’, future research must directly confront the concrete deficiencies identified in this review. Based on the critical gaps in the current evidence-specifically, the absence of head-to-head comparisons between the CGT flavonoid fraction and its major individual constituents, the lack of standardized and chemically characterized CGT preparations, and the paucity of direct target engagement studies-we propose three prioritized research priorities.

1. Empirical Validation of the Synergy Hypothesis: Current claims of synergy among CGT flavonoids remain hypothetical. The most immediate need is a head-to-head comparative study in an established MASLD model (e.g., HFD-fed mice) testing the efficacy of a chemically standardized CGT flavonoid fraction against equivalent doses of its three major individual constituents (naringin, neohesperidin, rhoifolin) and against rationally designed combinations. Such a study would resolve whether the fraction offers any advantage over individual compounds and provide a rational basis for further development (Table 8).
2. Direct Target Deconvolution: Most mechanistic inferences are based on pathway inhibition assays without evidence of direct target engagement. Given the PAINS concerns associated with flavonoids, future studies must employ chemical proteomics (e.g., affinity-based pull-down coupled with mass spectrometry) and cellular target engagement assays (e.g., CETSA) to identify the direct protein targets of naringin, neohesperidin, and rhoifolin within immune and metabolic signaling networks. This is essential to distinguish specific pharmacology from non-specific redox effects.
3. Standardized Preparation and PK-PD Modeling: The absence of a widely accepted, chemically standardized CGT preparation hampers reproducibility and translational progress. A priority should be the development and characterization of a GMP-grade CGT extract with quantified content of naringin, neohesperidin, and rhoifolin, followed by detailed ADME studies in MASLD-relevant animal models. These studies must measure parent flavonoids and their key metabolites in plasma, liver tissue, and immune cells, and correlate these exposures with pharmacodynamic markers (e.g., p-NF-κB, cytokine levels) to establish PK-PD relationships that can guide clinical dosing.

**TABLE 8:** Validation status of proposed mechanisms for CGT flavonoids in MASLD: Direct binding evidence *versus* indirect assays.

Proposed mechanism	Pathway component	Tier 1/2 evidence in MASLD models?	Direct binding evidence (CETSA, SPR, ITC, Kd)?	Assay type in source studies	PAINS risk assessment	Validation status
NF-κB pathway inhibition	IKK, IκB, p65	No	None (0 studies)	Western blot (p-IκB, p-p65), ELISA (TNF-α, IL-6)	High	Unvalidated - indirect only
MAPK pathway inhibition	JNK, p38	No	None (0 studies)	Western blot (p-JNK, p-p38) in non-MASLD models (colitis, lung injury)	High	Unvalidated - indirect only
JAK-STAT pathway inhibition	JAK1, JAK2, STAT1, STAT3	No	None (0 studies)	Western blot (p-JAK, p-STAT) in non-MASLD models (autoimmune hepatitis, microglia)	High	Unvalidated - indirect only
Macrophage M1→M2 polarization	CD86, CD206, Arg1, iNOS	No	None (0 studies)	No flow cytometry data in hepatic macrophages	N/A	No evidence - hypothetical
Nrf2 antioxidant activation	Nrf2, KEAP1, HO-1, NQO1	Partial (Tier 2 in MASLD)	None (0 studies)	Western blot (nuclear Nrf2), qPCR (HO-1, NQO1)	Medium	Indirect - requires CETSA

Only after these foundational gaps are addressed will the field be positioned to justify progression to human trials, which should follow a staged approach beginning with Phase I safety and pharmacokinetic studies in healthy volunteers, followed by biomarker-rich Phase IIa proof-of-concept trials in well-characterized MASLD patients.

### 6.4 Drug-drug interaction (DDI) risks and CYP enzyme considerations

A critical safety consideration omitted from previous reviews is the potential for CGT flavonoids to interact with cytochrome P450 (CYP) enzymes, leading to altered pharmacokinetics of co-administered medications. MASLD patients frequently receive polypharmacy for comorbidities including type 2 diabetes (metformin, sulfonylureas, SGLT2 inhibitors, GLP-1 agonists), dyslipidemia (statins, ezetimibe, fibrates), hypertension (ACE inhibitors, ARBs, calcium channel blockers), and cardiovascular disease (aspirin, clopidogrel). Table 9 summarizes known CYP interactions for major CGT-characteristic flavonoids.

**TABLE 9:** Cytochrome P450 interactions for CGT-characteristic flavonoids and potential drug-drug interaction risks.

Flavonoid	CYP enzyme	IC50/Ki/Km (μM)	Effect type	FDA perpetrator classification	Potential DDI risk	Reference(s)
Naringin (parent glycoside)	CYP3A4	Inhibition IC50 ∼50–100 μM	Inhibition (weak)	Class 2 (moderate)	May increase statin (atorvastatin, simvastatin) exposure	(Xiao et al., 2024; Ganesan and Xu, 2018)
Naringenin (aglycone)	CYP3A4	Inhibition IC50 ∼10–30 μM; Induction Km ∼10 μM	Mixed inhibition/induction	Class 2–3	Complex effects on statins, nifedipine	(Kim et al., 2009; Wang et al., 2020)
Naringenin	CYP1A2	Inhibition IC50 ∼20–50 μM	Inhibition (weak)	Class 3	Potential caffeine interaction	Kim et al. (2009)
Naringenin	CYP2D6	Inhibition IC50 ∼15–40 μM	Inhibition (weak-moderate)	Class 2	May affect metoprolol, carvedilol, codeine metabolism	Wang et al. (2020)
Neohesperidin	CYP3A4	Inhibition IC50 ∼30–80 μM	Inhibition (weak)	Class 3	Low clinical concern	(Deng et al., 2022; Lee et al., 2004)
Hesperetin	CYP3A4	Inhibition IC50 ∼40–90 μM	Inhibition (weak)	Class 3	Low clinical concern	(Li et al., 2024; Li et al., 2021)
Hesperetin	CYP2D6	Inhibition IC50 ∼25–60 μM	Inhibition (weak)	Class 3	Potential but low risk	Li et al. (2021)

Critical DDI considerations for clinical translation: (1) Statin interaction risk: The inhibition of CYP3A4 by naringenin (IC50 ∼10–30 μM) could theoretically increase systemic exposure of CYP3A4-metabolized statins (atorvastatin, simvastatin, lovastatin), potentially increasing risk of statin-associated muscle symptoms or rhabdomyolysis. However, given that achievable plasma concentrations of free aglycones are typically <1 μM (Table 7), the clinical relevance of this interaction is uncertain without dedicated DDI studies. (2) Antiplatelet agent considerations: CYP2C19 and CYP3A4 metabolize clopidogrel to its active metabolite. CYP inhibition could theoretically reduce antiplatelet efficacy, though data for CGT flavonoids on CYP2C19 are lacking. (3) Antidiabetic agent interactions: While direct CYP-mediated interactions with metformin (primarily renally excreted) or SGLT2 inhibitors are unlikely, naringenin has been reported to inhibit organic cation transporter 2 (OCT2) and multidrug and toxin extrusion proteins (MATEs) *in vitro*, potentially affecting metformin disposition. This requires dedicated transporter interaction studies. (4) FDA guidance: Based on the FDA’s Guidance for Industry on Drug Interaction Studies (2020), the concentrations required for clinically relevant CYP inhibition are typically >100-fold below the therapeutic plasma concentration of the perpetrator. Given that achievable plasma concentrations of CGT flavonoid aglycones are <1 μM and CYP inhibition IC50 values are >10 μM (ratio <0.1), the risk of clinically significant DDI is low but cannot be excluded without dedicated clinical DDI studies. We strongly recommend that future Phase I trials include a dedicated DDI sub-study with a sensitive CYP3A4 probe substrate (e.g., midazolam) and assessment of statin pharmacokinetics before CGT flavonoids are advanced to Phase II/III trials in polypharmacy MASLD populations.

## 7 Conclusion

In conclusion, preclinical evidence suggests that flavonoids from C. grandis ‘Tomentosa’ (CGT) can ameliorate key features of MASLD, including hepatic steatosis, inflammation, and oxidative stress. Regarding the purported mechanisms, it is essential to recognize that the current evidence for modulation of immune-inflammatory signaling pathways (NF-κB, MAPK, JAK-STAT) and immune cell functions (macrophage polarization, T-cell balance) is largely indirect, derived predominantly from studies using non-CGT preparations, non-MASLD models, or isolated compounds from other citrus species. Direct evidence for these mechanisms in MASLD-relevant hepatic immune cells using authenticated CGT extracts remains absent, and much of the available evidence is associative and may be subject to alternative interpretations, including potential assay artifacts.

The journey from promising preclinical data to clinical application for CGT flavonoids faces several hurdles. The observed bioactivities may be influenced by pan-assay interference compound (PAINS) artifacts, and the complete lack of direct target engagement studies leaves open the fundamental question of specificity *versus* general cytoprotection or redox modulation. Furthermore, the absence of studies examining CGT effects on Kupffer cell polarization or intrahepatic T-cell subsets in MASLD models means that claims of immunomodulation remain hypothetical at present. To bridge these gaps, future research must prioritize: (1) employing target deconvolution strategies (e.g., CETSA, DARTS): to identify direct molecular interactors of CGT flavonoids; (2) conducting rigorous comparative studies between the total flavonoid fraction and its major constituents to empirically test synergy hypotheses; and (3) establishing pharmacokinetic-pharmacodynamic relationships in relevant disease models. Furthermore, the field would benefit from consensus on chemically and biologically standardized CGT preparations.

Ultimately, the potential of CGT flavonoids must be tested in humans. We propose that future clinical research should progress in a stepwise manner: initial phase I/II trials assessing safety, tolerability, and pharmacokinetics of a well-characterized CGT extract in patients with MASLD, followed by randomized controlled trials employing robust endpoints such as MRI-PDFF for steatosis and ELF score or biopsy for fibrosis. By addressing the current mechanistic and translational gaps through a concerted and critical research effort, C. grandis ‘Tomentosa’ may evolve from a traditional remedy into a evidence-based therapeutic option for MASLD.

## Data Availability

The original contributions presented in the study are included in the article/Supplementary Material, further inquiries can be directed to the corresponding author.

## Supplementary material

SUPPLEMENTARY FIGURE S1 PRISMA 2020 flow diagram for the systematic search and selection process of studies on *Citrus grandis* ‘Tomentosa’ flavonoids in MASLD. The diagram outlines the four stages of the systematic review process-identification, screening, eligibility, and inclusion-based on the PRISMA 2020 framework. A total of 287 records were identified from PubMed, Web of Science, Scopus, and CNKI. After removing 94 duplicates, 193 records were screened. Based on title and abstract screening, 121 records were excluded (68 irrelevant to liver disease, 42 reviews/editorials/conference abstracts, 11 non-English/Chinese). The remaining 72 full-text articles were assessed for eligibility, of which 30 were excluded (18 lacking quantitative flavonoid data, 7 not specific to C. grandis ‘Tomentosa’, 5 without MASLD-relevant outcomes). Ultimately, 42 studies met all predefined inclusion criteria and were included in the qualitative synthesis, comprising 24 in vivo studies, 15 in vitro studies, and 3 clinical studies.

SUPPLEMENTARY FIGURE S2 Hypothesized multi-target modulation of immuneinflammatory signaling pathways by C. grandis ‘Tomentosa’ flavonoids (CGT) in MASLD. CRITICAL CAVEAT: This schematic is a hypothesis-generating model only. No direct binding evidence (CETSA, SPR, ITC, Kd determination) exists for any proposed target of CGT flavonoids. No co-IP, FRET, or CRISPR validation exists for any predicted protein-protein interaction shown in this figure. All network pharmacology predictions (depicted as dashed arrows) are derived from STRING/TCMSP databases and have not been experimentally validated in any MASLD model or with any CGT preparation. The vast majority of evidence for pathway modulation is derived from Tier 3 (non-CGT, non-MASLD) studies or from network pharmacology predictions that have not been experimentally validated in MASLD models using CGT preparations. Direct evidence for any of these pathways in MASLD-relevant hepatic immune cells (Kupffer cells, T cells) using authenticated CGT extracts is currently absent. The diagram depicts a standard immuneinflammatory signaling network including NF-κB, MAPK (JNK/p38), and JAK-STAT pathways. Red inhibitory symbols and “CGT Flavonoids” labels indicate points where experimental evidence from non-MASLD models or non-CGT preparations (e.g., Refs [61-65]) suggests potential inhibition (e.g., on IKK, JNK, p38, JAK phosphorylation); these do not represent validated direct target engagement. Solid arrows represent well-established canonical pathway connections (e.g., IKK→IκB→NF-κB, JAK→STAT) supported by general biochemical literature, not CGT-specific validation. Dashed arrows denote network pharmacology predictions that have received no experimental validation whatsoever. The dashed box at the bottom highlights the antioxidant properties of CGT, with a dotted arrow indicating that reduced ROS may indirectly suppress these pathways - a potential alternative explanation for observed effects that does not require direct target engagement. The dashed box at the top indicates proposed immunomodulatory effects on macrophage polarization (M1 to M2 shift); however, direct evidence for this effect in hepatic macrophages (Kupffer cells) in MASLD models using CGT preparations is currently lacking, and this aspect remains hypothetical. Readers are strongly cautioned that this entire figure should be interpreted as an unvalidated working hypothesis requiring extensive wet-lab confirmation (co-IP, FRET, CRISPR, CETSA, or SPR).

## References

[B1] Anmol R. J. Marium S. Hiew F. T. Han W. C. Kwan L. K. Wong A. K. Y. (2021). Phytochemical and therapeutic potential of citrus grandis (L.) osbeck: a review. J. Evid. Based Integr. Med. 26, 2515690X211043741. 10.1177/2515690x211043741 PMC8527587 34657477

[B2] Barrow F. Khan S. Wang H. Revelo X. S. (2021). The emerging role of B cells in the pathogenesis of NAFLD. Hepatology 74 (4), 2277–2286. 10.1002/hep.31889 33961302 PMC8463421

[B3] Benavente-García O. Castillo J. (2008). Update on uses and properties of citrus flavonoids: new findings in anticancer, cardiovascular, and anti-inflammatory activity. J. Agric. Food Chem. 56 (15), 6185–6205. 10.1021/jf8006568 18593176

[B4] Buzdağlı Y. Eyipınar C. D. Kacı F. N. Tekin A. (2023). Effects of hesperidin on anti-inflammatory and antioxidant response in healthy people: a meta-analysis and meta-regression. Int. J. Environ. Health Res. 33 (12), 1390–1405. 10.1080/09603123.2022.2093841 35762134

[B70] Cai Y. Ma W. Hou Y. Nisar M. F. Long Y. Chen B. (2025). Citri reticulatae pericarpium modulates gut microbiota: impacts on human health. Food Res. Int. 217, 116745. 10.1016/j.foodres.2025.116745 40597475

[B5] Cataldo I. Sarcognato S. Sacchi D. Cacciatore M. Baciorri F. Mangia A. (2021). Pathology of non-alcoholic fatty liver disease. Pathologica 113 (3), 194–202. 10.32074/1591-951x-242 34294937 PMC8299321

[B80] Cayetano-Salazar L. Olea-Flores M. Zuñiga-Eulogio M. D. Weinstein-Oppenheimer C. Fernández-Tilapa G. Mendoza-Catalán M. A. (2021). Natural isoflavonoids in invasive cancer therapy: From bench to bedside. Phytother. Res. 35 (8), 4092–4110. 10.1002/ptr.7072 33720455

[B6] Cesar T. B. Ramos F. M. M. Ribeiro C. B. (2022). Nutraceutical eriocitrin (eriomin) reduces hyperglycemia by increasing glucagon-like peptide 1 and downregulates systemic inflammation: a crossover-randomized clinical trial. J. Med. Food 25 (11), 1050–1058. 10.1089/jmf.2021.0181 35796695 PMC9700344

[B7] Chen J. Qin X. Chen M. Chen T. Chen Z. He B. (2024). Biological activities, molecular mechanisms, and clinical application of naringin in metabolic syndrome. Pharmacol. Res. 202, 107124. 10.1016/j.phrs.2024.107124 38428704

[B9] Cheng Y. Feng S. Sheng C. Yang C. Li Y. (2024). Nobiletin from citrus peel: a promising therapeutic agent for liver disease-pharmacological characteristics, mechanisms, and potential applications. Front. Pharmacol. 15, 1354809. 10.3389/fphar.2024.1354809 38487166 PMC10938404

[B10] Deng M. Zhang S. Dong L. Huang F. Jia X. Su D. (2022). Shatianyu (citrus grandis L. osbeck) flavonoids and dietary fiber in combination are more effective than individually in alleviating high-fat-diet-induced hyperlipidemia in mice by altering gut microbiota. J. Agric. Food Chem. 70 (46), 14654–14664. 10.1021/acs.jafc.2c03797 36322531

[B11] Efferth T. Koch E. (2011). Complex interactions between phytochemicals. The multi-target therapeutic concept of phytotherapy. Curr. Drug Targets 12 (1), 122–132. 10.2174/138945011793591626 20735354

[B12] Fang J. Yu C. H. Li X. J. Yao J. M. Fang Z. Y. Yoon S. H. (2022). Gut dysbiosis in nonalcoholic fatty liver disease: pathogenesis, diagnosis, and therapeutic implications. Front. Cell. Infect. Microbiol. 12, 997018. 10.3389/fcimb.2022.997018 36425787 PMC9679376

[B13] Ganesan K. Xu B. (2018). Anti-obesity effects of medicinal and edible mushrooms. Molecules 23 (11), 2880. 10.3390/molecules23112880 30400600 PMC6278646

[B14] Grander C. Grabherr F. Tilg H. (2023). Non-alcoholic fatty liver disease: pathophysiological concepts and treatment options. Cardiovasc Res. 119 (9), 1787–1798. 10.1093/cvr/cvad095 37364164 PMC10405569

[B15] Guo X. Yin X. Liu Z. Wang J. (2022). Non-alcoholic fatty liver disease (NAFLD) pathogenesis and natural products for prevention and treatment. Int. J. Mol. Sci. 23 (24), 15489. 10.3390/ijms232415489 36555127 PMC9779435

[B71] Guo X. F. Ruan Y. Li Z. H. Li D. (2019). Flavonoid subclasses and type 2 diabetes mellitus risk: a meta-analysis of prospective cohort studies. Crit. Rev. Food Sci. Nutr. 59 (17), 2850–2862. 10.1080/10408398.2018.1476964 29768032

[B16] Guo L. Ding Q.-Y. Duan W.-H. Guan Q. J. Ren Y. L. Xue Y. Z. (2026). Goji-derived exosomes-like nanoparticles ameliorate alcohol-induced acute liver injury by modulating gut microbiota and metabolites. Food and Med. Homol. 3 (1), 9420081. 10.26599/fmh.2026.9420081

[B17] Han S. K. Baik S. K. Kim M. Y. (2023). Non-alcoholic fatty liver disease: definition and subtypes. Clin. Mol. Hepatol. 29 (Suppl. l), S5–S16. 10.3350/cmh.2022.0424 36577427 PMC10029964

[B18] Hsu H. Sheth C. C. Veses V. (2021). Herbal extracts with antifungal activity against candida albicans: a systematic review. Mini Rev. Med. Chem. 21 (1), 90–117. 10.2174/1389557520666200628032116 32600229

[B19] Hua Y. Q. Zeng Y. Xu J. Xu X. L. (2021). Naringenin alleviates nonalcoholic steatohepatitis in middle-aged Apoe^-/-^mice: role of SIRT1. Phytomedicine 81, 153412. 10.1016/j.phymed.2020.153412 33234364

[B20] Huby T. Gautier E. L. (2022). Immune cell-mediated features of non-alcoholic steatohepatitis. Nat. Rev. Immunol. 22 (7), 429–443. 10.1038/s41577-021-00639-3 34741169 PMC8570243

[B21] Juanola O. Martínez-López S. Francés R. Gómez-Hurtado I. (2021). Non-alcoholic fatty liver disease: Metabolic, genetic, epigenetic and environmental risk factors. Int. J. Environ. Res. Public Health 18 (10), 5227. 10.3390/ijerph18105227 34069012 PMC8155932

[B22] Kim H. J. Song J. Y. Park H. J. Park H. K. Yun D. H. Chung J. H. (2009). Naringin protects against rotenone-induced apoptosis in human neuroblastoma SH-SY5Y cells. Korean J. Physiol. Pharmacol. 13 (4), 281–285. 10.4196/kjpp.2009.13.4.281 19885011 PMC2766708

[B24] Kong F. Ding Z. Zhang K. Duan W. Qin Y. Su Z. (2020). Optimization of extraction flavonoids from exocarpium citri grandis and evaluation its hypoglycemic and hypolipidemic activities. J. Ethnopharmacol. 262, 113178. 10.1016/j.jep.2020.113178 32736047

[B75] Kumar S. Pandey A. K. (2013). Chemistry and biological activities of flavonoids: an overview. Sci. World J., 162750. 10.1155/2013/162750 24470791 PMC3891543

[B25] Lee N. K. Choi S. H. Park S. H. Park E. K. Kim D. H. (2004). Antiallergic activity of hesperidin is activated by intestinal microflora. Pharmacology 71 (4), 174–180. 10.1159/000078083 15240993

[B26] Lee G. H. Peng C. Park S. A. Hoang T. H. Lee H. Y. Kim J. (2020). Citrus peel extract ameliorates high-fat diet-induced NAFLD *via* activation of AMPK signaling. Nutrients 12 (3), 673. 10.3390/nu12030673 32121602 PMC7146518

[B27] Li J. Wang T. Liu P. Yang F. Wang X. Zheng W. (2021). Hesperetin ameliorates hepatic oxidative stress and inflammation *via* the PI3K/AKT-Nrf2-ARE pathway in oleic acid-induced HepG2 cells and a rat model of high-fat diet-induced NAFLD. Food Funct. 12 (9), 3898–3918. 10.1039/d0fo02736g 33977953

[B28] Li J. Deng S. H. Li J. Li L. Zhang F. Zou Y. (2022). Obacunone alleviates ferroptosis during lipopolysaccharide-induced acute lung injury by upregulating Nrf2-dependent antioxidant responses. Cell. Mol. Biol. Lett. 27 (1), 29. 10.1186/s11658-022-00318-8 35305560 PMC8933916

[B29] Li L. Liu R. He J. Li J. Guo J. Chen Y. (2022). Naringin regulates microglia BV-2 activation and inflammation *via* the JAK/STAT3 pathway. Evid. Based Complement. Altern. Med. 2022, 3492058. 10.1155/2022/3492058 PMC9135528 35646153

[B30] Li W. Cai Z. Schindler F. Afjehi-Sadat L. Montsch B. Heffeter P. (2024). Elevated PINK1/Parkin-Dependent mitophagy and boosted mitochondrial function mediate protection of HepG2 cells from excess palmitic acid by hesperetin. J. Agric. Food Chem. 72 (23), 13039–13053. 10.1021/acs.jafc.3c09132 38809522 PMC11181321

[B32] Liu J. (2022). Naringenin alleviates nonalcoholic steatohepatitis by modulating hepatic lipid metabolism and inflammation. Biomed. Pharmacother. 146, 112442. 35062053

[B33] Liu J. Ding M. Bai J. Luo R. Liu R. Qu J. (2023). Decoding the role of immune T cells: a new territory for improvement of metabolic-associated fatty liver disease. Imeta 2 (1), e76. 10.1002/imt2.76 38868343 PMC10989916

[B34] Lombardo G. E. Navarra M. Cremonini E. (2024). A flavonoid-rich extract of bergamot juice improves high-fat diet-induced intestinal permeability and associated hepatic damage in mice. Food Funct. 15 (19), 9941–9953. 10.1039/d4fo02538e 39263833

[B78] Lu J. Chen J. Li S. Y. Pan G. J. Ou Y. Yuan L. F. (2024). Naringin and naringenin: potential multi-target agents for Alzheimer’s disease. Curr. Med. Sci. 44 (5), 867–882. 10.1007/s11596-024-2921-z 39347923

[B35] Lu K. Yip Y. M. (2023). Therapeutic potential of bioactive flavonoids from citrus fruit peels toward obesity and diabetes mellitus. Future Pharmacol. 3, 14–37. 10.3390/futurepharmacol3010002

[B72] Ming L. G. Chen K. M. Xian C. J. (2013). Functions and action mechanisms of flavonoids genistein and icariin in regulating bone remodeling. J. Cell Physiol. 228 (3), 513–521. 10.1002/jcp.24158 22777826

[B36] Najar I. A. Bhat M. H. Qadrie Z. L. Amaldoss M. J. N. Kushwah A. S. Singh T. G. (2022). Cardioprotection by citrus grandis (L.) peel ethanolic extract in alloxan-induced cardiotoxicity in diabetic rats. Biomed. Res. Int. 2022, 2807337. 10.1155/2022/2807337 35757467 PMC9225855

[B37] Nassir F. (2022). NAFLD: mechanisms, treatments, and biomarkers. Biomolecules 12 (6), 824. 10.3390/biom12060824 35740949 PMC9221336

[B38] Notarnicola M. Tutino V. De Nunzio V. Cisternino A. M. Cofano M. Donghia R. (2024). Daily Orange consumption reduces hepatic steatosis prevalence in patients with metabolic dysfunction-associated steatotic liver disease: exploratory outcomes of a randomized clinical trial. Nutrients 16 (18), 3191. 10.3390/nu16183191 39339791 PMC11435367

[B39] Pafili K. Roden M. (2021). Nonalcoholic fatty liver disease (NAFLD) from pathogenesis to treatment concepts in humans. Mol. Metab. 50, 101122. 10.1016/j.molmet.2020.101122 33220492 PMC8324683

[B41] Paternostro R. Trauner M. (2022). Current treatment of non-alcoholic fatty liver disease. J. Intern Med. 292 (2), 190–204. 10.1111/joim.13531 35796150 PMC9546342

[B42] Powell E. E. Wong V. W. Rinella M. (2021). Non-alcoholic fatty liver disease. Lancet 397 (10290), 2212–2224. 10.1016/s0140-6736(20)32511-3 33894145

[B79] Ramaiah P. Baljon K. J. Hjazi A. Qasim M. T. Salih Al-Ani O. A. Imad S. (2024). Dietary polyphenols and the risk of metabolic syndrome: a systematic review and meta-analysis. BMC Endocr. Disord. 24 (1), 26. 10.1186/s12902-024-01556-x 38429765 PMC10905819

[B43] Raza S. Rajak S. Upadhyay A. Tewari A. Anthony Sinha R. (2021). Current treatment paradigms and emerging therapies for NAFLD/NASH. Front. Biosci. Landmark Ed. 26 (2), 206–237. 10.2741/4892 33049668 PMC7116261

[B44] Sacks D. Baxter B. Campbell B. C. V. Carpenter J. S. Cognard C. Dippel D. (2018). Multisociety consensus quality improvement revised consensus statement for endovascular therapy of acute ischemic stroke. Int. J. Stroke 13 (6), 612–632. 10.3174/ajnr.a5638 29786478

[B45] Saiman Y. Duarte-Rojo A. Rinella M. E. (2022). Fatty liver disease: diagnosis and stratification. Annu. Rev. Med. 73, 529–544. 10.1146/annurev-med-042220-020407 34809436 PMC10074159

[B46] Singh B. Singh J. P. Kaur A. Singh N. (2020). Phenolic composition, antioxidant potential and health benefits of citrus peel. Food Res. Int. 132, 109114. 10.1016/j.foodres.2020.109114 32331689

[B73] Sudhakaran M. Doseff A. I. (2020). The targeted impact of flavones on obesity-induced inflammation and the potential synergistic role in cancer and the gut microbiota. Molecul. 27 (11), 2477. 10.3390/molecules25112477 32471061 PMC7321129

[B47] Targher G. Tilg H. Byrne C. D. (2021). Non-alcoholic fatty liver disease: a multisystem disease requiring a multidisciplinary and holistic approach. Lancet Gastroenterol. Hepatol. 6 (7), 578–588. 10.1016/s2468-1253(21)00020-0 33961787

[B48] Tilg H. Adolph T. E. Dudek M. Knolle P. (2021). Non-alcoholic fatty liver disease: the interplay between metabolism, microbes and immunity. Nat. Metab. 3 (12), 1596–1607. 10.1038/s42255-021-00501-9 34931080

[B49] Tocmo R. Pena-Fronteras J. Calumba K. F. Mendoza M. Johnson J. J. (2020). Valorization of pomelo (citrus grandis osbeck) peel: a review of current utilization, phytochemistry, bioactivities, and mechanisms of action. Compr. Rev. Food Sci. Food Saf. 19 (4), 1969–2012. 10.1111/1541-4337.12561 33337092

[B77] Tsai J. Y. Hsu H. C. Tai C. J. Wu C. C. (2025). 7,4′-dimethoxy-3-hydroxyflavone, a protease-activated receptor 4 (PAR4) inhibitor with antioxidant activity, ameliorates diabetic endothelial dysfunction. Br J. Pharmacol. 182 (19), 4592–4610. 10.1111/bph.70105 40501374

[B50] Visnagri A. Kandhare A. D. Chakravarty S. Ghosh P. Bodhankar S. L. (2014). Hesperidin, a flavanoglycone attenuates experimental diabetic neuropathy *via* modulation of cellular and biochemical marker to improve nerve functions. Pharm. Biol. 52 (7), 814–828. 10.3109/13880209.2013.870584 24559476

[B51] Walsh S. K. Pettigrew K. Mezzani I. Alaswad I. Bermano G. (2025). Role of selenium and 17β oestradiol in modulating lipid accumulation in *in vitro* models of obesity and NAFLD. Food and Med. Homol. 2 (4), 9420056. 10.26599/fmh.2025.9420056

[B52] Wang Q. Ou Y. Hu G. Wen C. Yue S. Chen C. (2020). Naringenin attenuates non-alcoholic fatty liver disease by down-regulating the NLRP3/NF-κB pathway in mice. Br. J. Pharmacol. 177 (8), 1806–1821. 10.1111/bph.14938 31758699 PMC7070172

[B53] Wang J. Li T. Cai H. Jin L. Li R. Shan L. (2021). Protective effects of total flavonoids from Qu Zhi Qiao (fruit of citrus paradisi cv. changshanhuyou) on OVA-induced allergic airway inflammation and remodeling through MAPKs and Smad2/3 signaling pathway. Biomed. Pharmacother. 138, 111421. 10.1016/j.biopha.2021.111421 33752061

[B55] Wang J. Yao Y. Yao T. Shi Q. Zeng Y. Li L. (2024). Hesperetin alleviated experimental colitis *via* regulating ferroptosis and gut microbiota. Nutrients 16 (14), 2343. 10.3390/nu16142343 39064786 PMC11279615

[B57] Wei S. Wang L. Evans P. C. Xu S. (2024). NAFLD and NASH: etiology, targets and emerging therapies. Drug Discov. Today 29 (3), 103910. 10.1016/j.drudis.2024.103910 38301798

[B58] Wu D. Liu L. Jiao N. Zhang Y. Yang L. Tian C. (2022). Targeting keystone species helps restore the dysbiosis of butyrate-producing bacteria in nonalcoholic fatty liver disease. Imeta 1 (4), e61. 10.1002/imt2.61 38867895 PMC10989787

[B59] Xiao P. Qu J. Wang Y. Fang T. Xiao W. Wang Y. (2024). Transcriptome and metabolome atlas reveals contributions of sphingosine and chlorogenic acid to cold tolerance in citrus. Plant Physiol. 196 (1), 634–650. 10.1093/plphys/kiae327 38875157

[B61] Yang X. Deng Y. Tu Y. Feng D. Liao W. (2022). Nobiletin mitigates NAFLD *via* lipophagy and inflammation. Food Funct. 13 (19), 10186–10199. 10.1039/d2fo01682f 36111578

[B62] Yang J. W. Zou Y. Chen J. Cui C. Song J. Yang M. M. (2023). Didymin alleviates metabolic dysfunction-associated fatty liver disease (MAFLD) *via* the stimulation of Sirt1-mediated lipophagy and mitochondrial biogenesis. J. Transl. Med. 21 (1), 921. 10.1186/s12967-023-04790-4 38115075 PMC10731721

[B74] Yang S. Wei Z. Luo J. Wang X. Chen G. Guan X. (2024). Integrated bioinformatics and multiomics reveal Liupao tea extract alleviating NAFLD via regulating hepatic lipid metabolism and gut microbiota. Phytomedicine 132, 155834. 10.1016/j.phymed.2024.155834 38941818

[B63] Yeh T. S. Yuan C. Ascherio A. Rosner B. A. Willett W. C. Blacker D. (2021). Long-term dietary flavonoid intake and subjective cognitive decline in US men and women. Neurology 97 (10), e1041–e1056. 10.1212/wnl.0000000000012454 34321362 PMC8448553

[B64] Zhang J. Lei H. Hu X. Dong W. (2020). Hesperetin ameliorates DSS-induced colitis by maintaining the epithelial barrier *via* blocking RIPK3/MLKL necroptosis signaling. Eur. J. Pharmacol. 873, 172992. 10.1016/j.ejphar.2020.172992 32035144

[B67] Zhou J. Zhou F. Wang W. Zhang X. J. Ji Y. X. Zhang P. (2020). Epidemiological features of NAFLD from 1999 to 2018 in China. Hepatology 71 (5), 1851–1864. 10.1002/hep.31150 32012320

[B69] Zhu Q. Jiang Y. Lin W. Gao M. Chen X. Li X. (2024a). Naringin as a natural candidate for anti-autoimmune hepatitis: inhibitory potency and hepatoprotective mechanism. Phytomedicine 129, 155722. 10.1016/j.phymed.2024.155722 38733905

[B76] Zhu M. Sun Y. Su Y. Guan W. Wang Y. Han J. (2024b). Luteolin: a promising multifunctional natural flavonoid for human diseases. Phytother. Res. 38 (7), 3417–3443. 10.1002/ptr.8217 38666435

