# Supplementary figures and images for: Therapeutic potential and mechanisms of flavonoids from Citrus grandis ‘Tomentosa’ in metabolic dysfunction-associated steatotic liver disease: a focus on immune-inflammatory signaling pathways
Source: Front Pharmacol. 2026 Jun 23;17:1825982. doi: 10.3389/fphar.2026.1825982 (PMC13338618; doi:10.3389/fphar.2026.1825982)

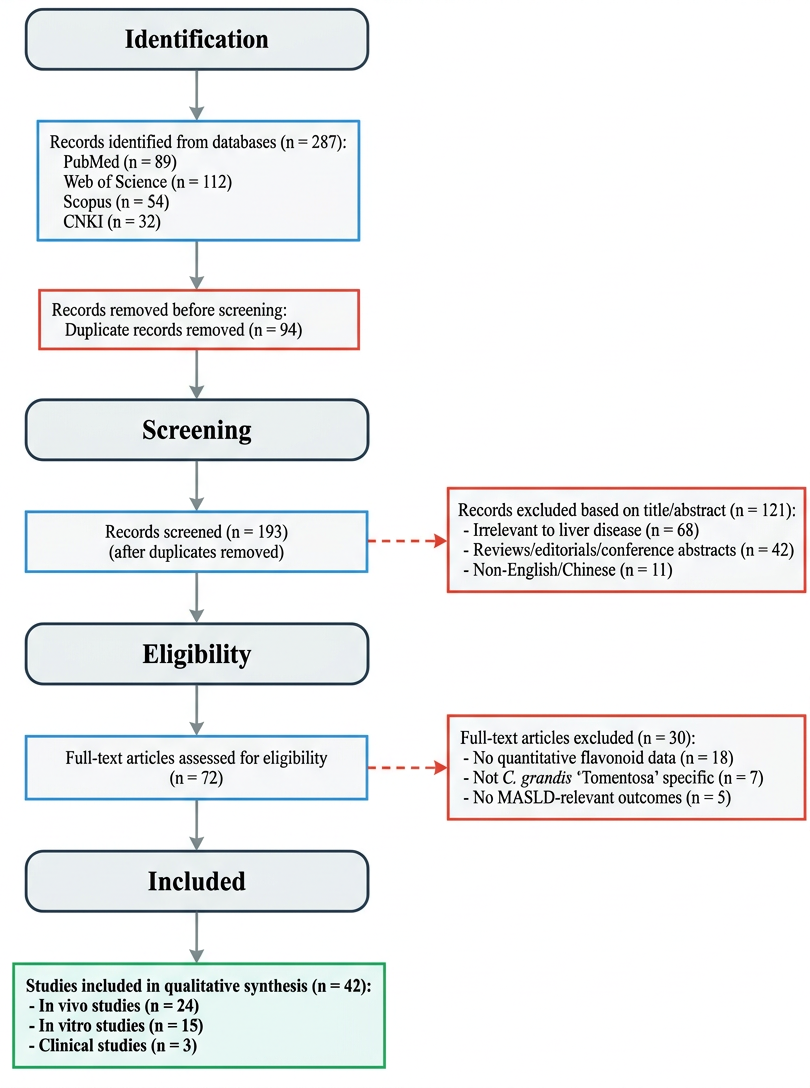

Supplement: Supplementary file 1 [file Image1.tiff]

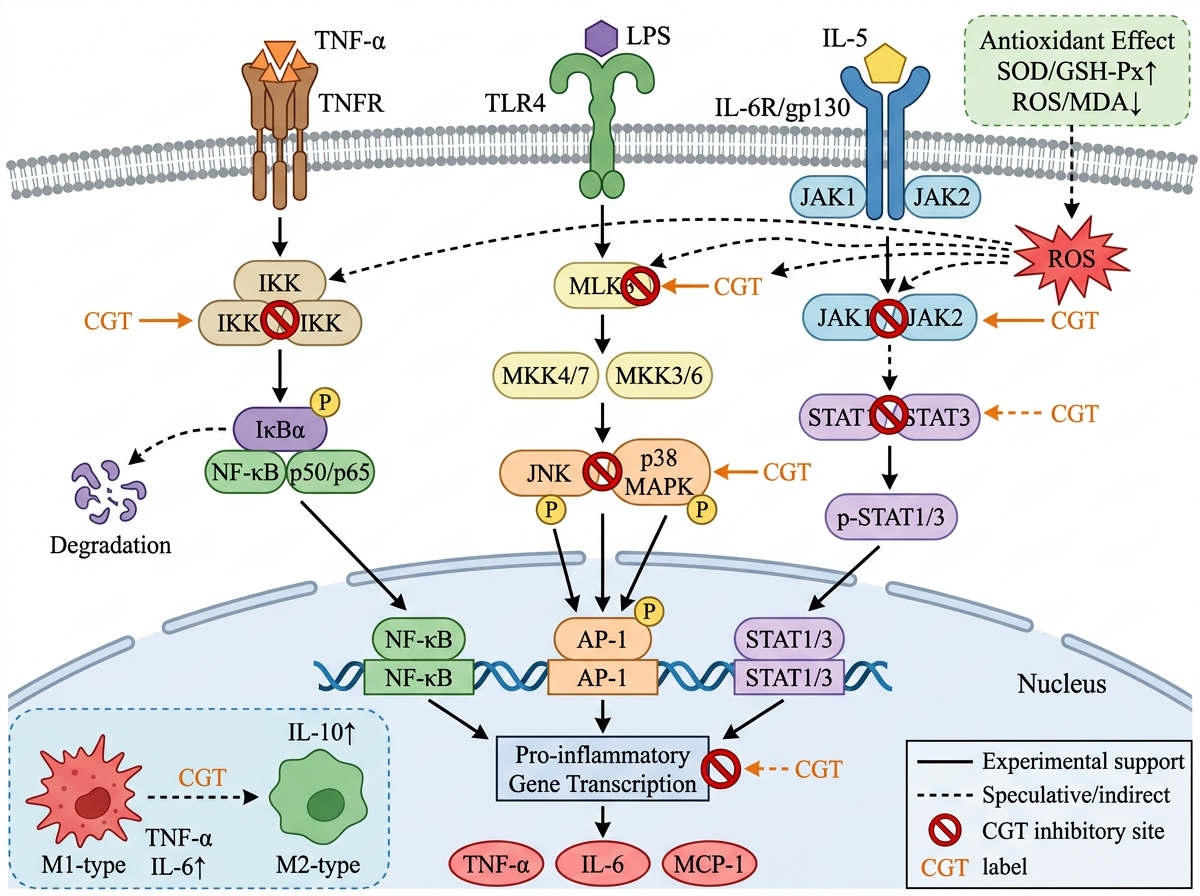

Supplement: Supplementary file 2 [file Image2.tiff]
